# Therapeutic Repurposing of Avanafil Against Lipopolysaccharide-induced Depression and Autoimmune Hepatitis: Gut-brain-liver Axis Orchestration Via Regulation of TLR4/NF-κB/IDO and Nrf2/HO-1 Pathways

**DOI:** 10.1007/s12035-026-05854-4

**Published:** 2026-04-25

**Authors:** Kawther Magdy Ibrahim, Hebatalla I. Ahmed, Laila A. Ramadan, Amany Balah

**Affiliations:** 1 Department of Pharmacology & Toxicology, Faculty of Pharmacy, Egyptian Russian University, Cairo, Egypt; 2 Department of Pharmacology & Toxicology, Faculty of Pharmacy, Al-Azhar University, Cairo, Egypt

**Keywords:** TLR4, Avanafil, Lipopolysaccharide, Leaky gut, Major depressive disorder, Autoimmune hepatitis

## Abstract

Emerging evidence has highlighted the gut-brain axis as a critical mediator in the pathogenesis of both major depressive disorder (MDD) and autoimmune hepatitis (AIH), where systemic inflammation and gut barrier dysfunction play pivotal roles. This study investigated the therapeutic potential of avanafil (AVA), a selective phosphodiesterase-5 inhibitor (PDE5I), in a lipopolysaccharide (LPS)–induced rat model that mimics inflammation-driven MDD and AIH. LPS administration significantly impaired cognitive behavior, induced depressive-like symptoms, elevated pro-inflammatory cytokines, disrupted gut and blood-brain barrier (BBB) integrity, and caused hepatic dysfunction. AVA treatment markedly improved behavioral performance in the novel object recognition and forced swim test, enhanced zonula occludens-1 (ZO-1) expression, and attenuated LPS-induced elevations of matrix metalloproteinase-9 (MMP-9), interleukin-1β (IL-1β), tumor necrosis factor alpha (TNF-α), and interleukin-6 (IL-6). Mechanistically, AVA downregulated the TLR4/NF-κB/IDO pathway, restored serotonin levels, reduced quinolinic acid accumulation, and activated the Nrf2/HO-1 signaling cascade in both hippocampal and liver tissues. These findings suggest that AVA exerts neuroprotective and hepatoprotective effects by modulating intestinal permeability, inflammation, and oxidative stress, making it a promising therapeutic candidate for conditions associated with systemic inflammation such as MDD and AIH.

**Graphical Abstract:**

Created in BioRender. Elgindy, A. (2026) https://BioRender.com/7czd3fv

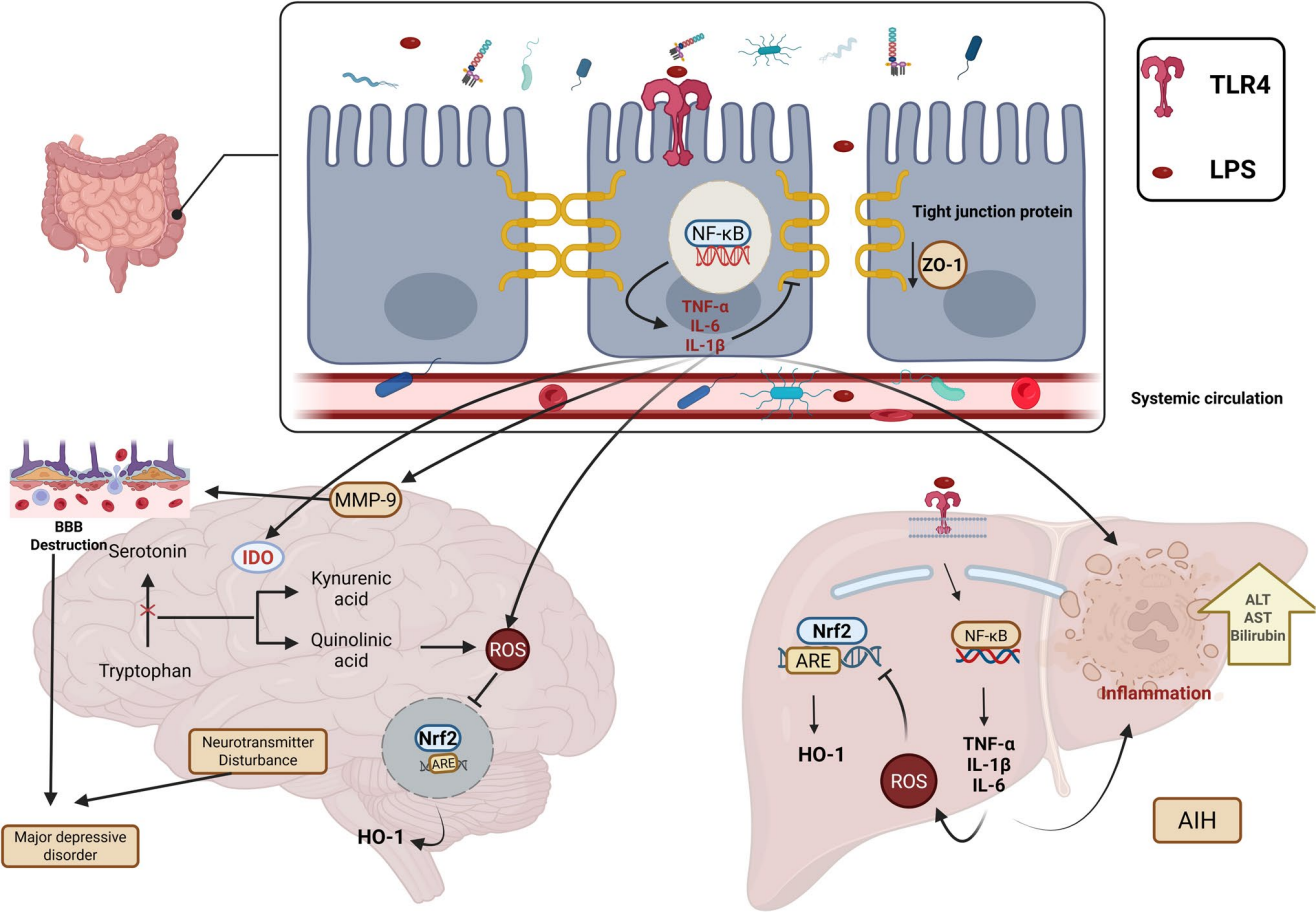

**Supplementary Information:**

The online version contains supplementary material available at 10.1007/s12035-026-05854-4.

## Introduction

The intestine is an important immunological organ that influences immune homeostasis, and maintaining gut homeostasis is essential for overall health. It plays a key role in immune regulation, preservation of barrier integrity, and maintenance of microbial balance [1]. Dysbiosis, or alterations in the composition of the gut microbiome, may promote the production of lipopolysaccharides (LPS) and thereby trigger inflammatory responses [2]. LPS, a glycolipid component of the outer membrane of Gram negative bacteria, acts as a potent endotoxin that vigorously activates innate immune cells, stimulates the release of multiple pro-inflammatory cytokines, thereby inducing injury in numerous organs, including the brain [3], and its role in the alteration of the intestinal tight junction (TJ) barrier has been well established [4].

A growing body of research supports a connection between the gut-brain axis and these inflammation-driven conditions. Compromise of intestinal barrier integrity (leaky gut) facilitates the translocation of bacterial endotoxins into the systemic compartment, such as LPS which exacerbates inflammation and accelerates the progression of both major depressive disorder (MDD) [5, 6] and autoimmune hepatitis (AIH) [7]. Furthermore, there is considerable evidence to show a bidirectional relationship between autoimmune diseases and depression, where the presence of one condition increases the risk of developing the other [8, 9].

Interestingly, Toll-like receptors (TLRs) regulate the bidirectional communication between bacteria and the immune system [6]. TLR4, a crucial pathogen recognition receptor, is activated by LPS. TLR4 activates the NF-κB signaling cascade by recruiting lipid rafts and interacting with adaptor molecules in response to stimulation. This activation initiates the release of pro-inflammatory cytokines, which encompass interleukin-6 (IL-6), tumor necrosis factor alpha (TNF-α), and interleukin-1β (IL-1β) [10], which are implicated in both neuroinflammation [11] and liver inflammation [12]. In the brain, these inflammatory mediators stimulate indoleamine-2,3-dioxygenase (IDO) [13], which drives the kynurenine pathway and diverts tryptophan metabolism from serotonin (5-HT) toward the production of neurotoxic metabolites such as quinolinic acid (QUIN) [14, 15] This shift also promotes the creation of reactive oxygen species (ROS), contributing to oxidative stress and neuronal degeneration in the hippocampus, which may further aggravate MDD [16]. Furthermore, heightened concentrations of pro-inflammatory mediators, notably TNF-α and IL-1β, disrupt the integrity of the blood-brain barrier (BBB), facilitating the influx of inflammatory mediators into the brain and initiating neuroinflammatory processes [17]. BBB disruption is a key mechanism in depression and other CNS disorders, including epilepsy and schizophrenia [3].

Hepatic immune balance depends on the coordinated activity of the intestinal barrier and the hepatic detoxification system, a relationship referred to as the gut-liver axis. When this balance is disturbed, microbial components such as LPS can leak through the intestinal wall into the portal circulation and reach the liver [7]. LPS in the liver triggers TLR4 on Kupffer cells and hepatocytes, prompting cytokine release and ROS generation [18]. When LPS concentrations exceed the detoxification capacity of the liver, chronic inflammation and cellular injury may occur, contributing to the pathogenesis of autoimmune hepatitis (AIH) [12, 19].

Molecular mimicry is another key mechanism linking LPS exposure to autoimmune liver disease. It occurs when bacterial antigens, such as LPS, share structural similarities with self-antigens in hepatocytes, leading to the activation of autoreactive T cells. This cross-reactivity elicits an immune response against liver tissues, driving autoimmune-mediated liver damage [8, 9]. Autoantibodies, particularly anti-nuclear antibodies (ANAs), are crucial for diagnosing and classifying type 1 AIH, as their levels correlate with inflammation and fibrosis, serving as valuable diagnostic and monitoring markers [20, 21].

Oxidative stress, a well-known cause of organ damage, is another pathogenic mechanism that occurs together with inflammation [12]. The transcription factor, nuclear factor erythroid 2–related factor 2 (Nrf2), acts as a master regulator, orchestrating the activation of genes involved in antioxidant and anti-inflammatory responses, thereby shielding cells from oxidative damage and inflammatory stress [22]. In recent years, growing evidence has implicated Nrf2 in the pathophysiology of several neuropsychiatric disorders, including MDD [23] and autoimmune disease such as AIH [12].

These experimental findings may also be viewed in light of clinical observations in AIH. Although direct clinical studies specifically diagnosing MDD in AIH remain limited, published patient cohorts consistently report a substantial burden of depressive symptoms and impaired mental well-being. For example, Schramm et al. [24] described impaired health-related quality of life together with depression and anxiety in patients with AIH, while Sockalingam et al. [25] reported that higher depressive and anxiety symptom scores were associated with poorer immunosuppressive adherence and treatment non-response. More recently, a large multicenter European study involving 882 patients with AIH confirmed that depression and anxiety were strongly associated with impaired health-related quality of life [26]. Collectively, these clinical data support the relevance of psychological burden in AIH and reinforce the clinical interest in inflammatory mechanisms shared with depression.

Avanafil, belonging to the second generation of phosphodiesterase-5 inhibitors (PDE5Is), is widely utilized as a standard treatment option for erectile dysfunction [27, 28]. Beyond its primary indication, PDE5I exhibits notable anti-inflammatory and antioxidant activities that enhance its therapeutic potential in inflammation-related disorders [29]. By selectively targeting cyclic guanosine monophosphate (cGMP), it exerts potent anti-inflammatory effects [30], while also functioning as an antioxidant by inhibiting free radical production and inducing heme oxygenase-1 (HO-1) expression [31]. Moreover, AVA demonstrates the highest permeability across the BBB, further supporting its promise as a neuroprotective agent [32].

Treatments for AIH rely on immunosuppressive therapies like corticosteroids and azathioprine, or liver transplantation in severe cases. However, liver transplantation is limited by donor shortages, immunosuppression risks, and high costs [12]. Similarly, MDD treatments, particularly selective serotonin reuptake inhibitors (SSRIs), face low response rates, side effects, and variable patient outcomes [33]. These challenges highlight the urgent need to investigate a novel therapeutic strategy targeting the shared inflammatory mechanisms of MDD and AIH. By focusing on restoring gut integrity and reducing systemic inflammation, this approach seeks to provide a more effective and comprehensive treatment for both conditions, addressing the root cause rather than merely alleviating symptoms. Therefore, this study was designed to elucidate the potential protective actions of AVA in the context of LPS-evoked inflammatory responses, MDD, and AIH in rats by restoring gut-brain-liver axis integrity and modifying the TLR4/NF-κB/IDO and Nrf2/HO-1 pathways.

## Material and Methods

### Animals

A cohort of forty male Sprague Dawley rats, each weighing between 150 and 200 g, were acquired from Nile Company (Co.) for Pharmaceutical and Chemical Industries, Egypt, and then accommodated within the accredited experimental animal section of the Faculty of Pharmacy, Al-Azhar University, where they were fed a standard pelleted diet and allowed unrestricted access to water, then left for a 1-week acclimatization period before the initiation of the experimental procedures, and were performed in strict adherence to a protocol authorized by the Institutional Animal Ethics Committee (approval number: RECCLAR11).

### Drugs and Chemicals

Avanafil (SML2799), lipopolysaccharide from *E. coli* (L-2630, serotype O111:B4), and all additional reagents utilized in this study were procured in analytical purity purchased from Sigma-Aldrich Chemie GmbH (Munich, Germany). Both AVA and LPS were prepared by dissolving them in normal saline (0.9%) to achieve the required concentrations and administered to the animals via the appropriate routes as specified in the experimental protocol.

### Experimental Protocol

**Scheme 1:**
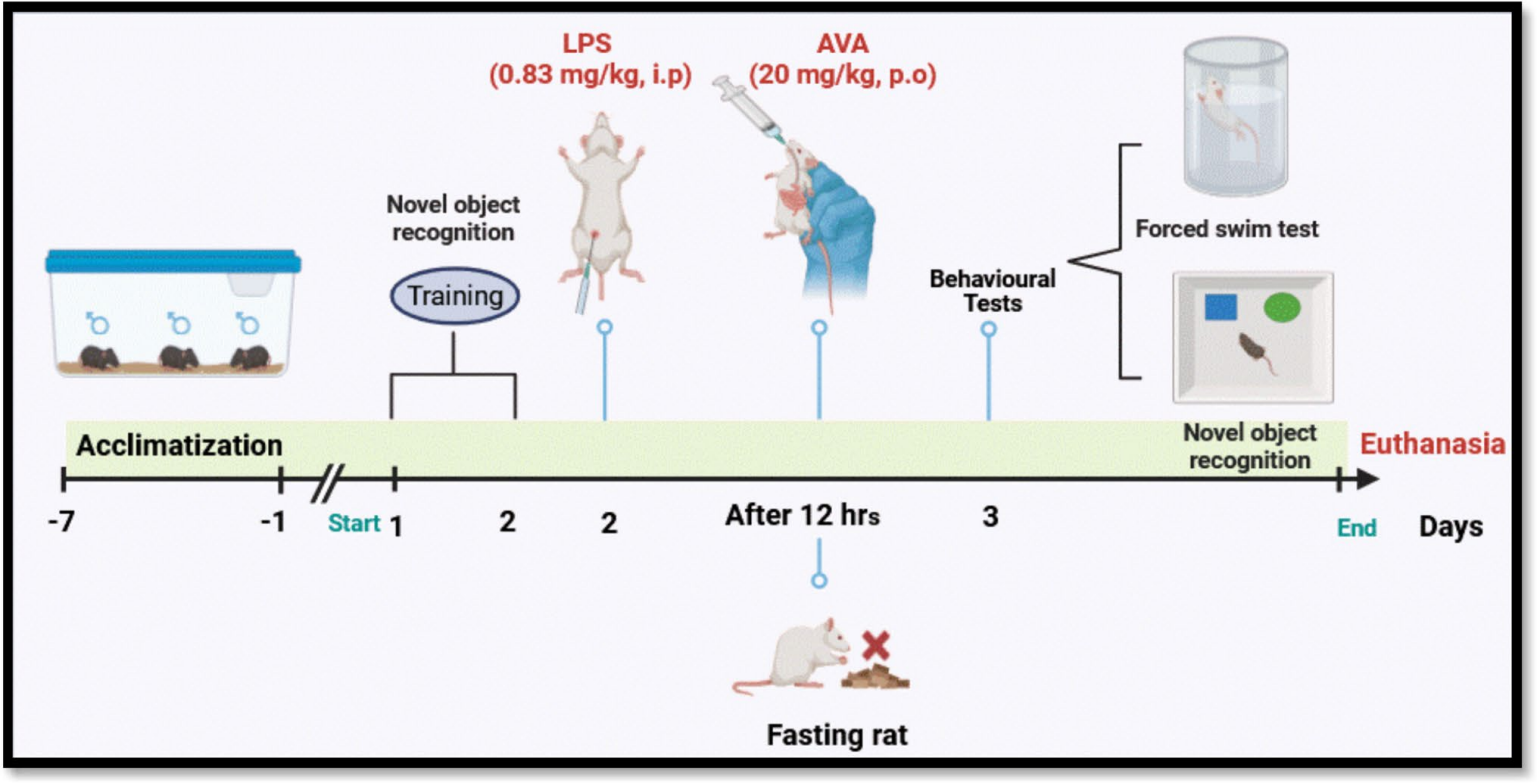
Timeline for LPS, AVA administration, and behavioral evaluations. Created in BioRender. Elgindy, A. (2026) https://BioRender.com/f3vtkgb

Forty rats were assigned at random to four groups (10 rats in each); animals in Group (I): acted as the control and were treated with (0.9%) normal saline. Group (II): animals received LPS (0.83 mg/kg, i.p) [34]. Group (III): rats were treated with avanafil (20 mg/kg, p.o), 12 h after LPS administration [35]. Group (IV): rats were administered avanafil (20 mg/kg, p.o) [35], after 12 h from normal saline (0.9%). The proposed protocol for behavioral evaluations and treatments is summarized in Scheme 1.

Following 24 h of LPS administration, all animals underwent a series of behavioral assessments as part of the experimental protocol. Animals were fasted overnight prior to the procedure, and blood specimens were collected from the retro-orbital venous plexus of experimental animals anesthetized with pentobarbital (50 mg/kg) [36]. Blood samples were centrifuged to isolate serum, which was then preserved at −20 °C for subsequent liver function analysis. At the same time, rats were euthanized by cervical dislocation. Tissue samples from the brain, liver, and colon were split into two portions: one portion was fixed in 10% neutral-buffered formalin for subsequent histopathological and immunohistochemical evaluation, while the other was preserved for biochemical assays, including enzyme-linked immunosorbent assay (ELISA), measurement of oxidative stress markers, and Western blotting.

### Behavioral Assessments

#### Novel Object Recognition Test

Test was executed according to previously established methods [37, 38]. Briefly, the procedure took place in a black open-field box (50 × 25 × 50 cm). Rats were allowed to explore the empty box for 10 min each day over 2 days of habituation. In the training phase, each rat was exposed to two identical objects placed about 30 cm apart within the box. At the start of the test day, one familiar object was replaced with a novel object, and the exploration of each object was recorded for 3 min using an overhead camera. Exploration was defined as the time an animal spent actively touching or sniffing an object. The discriminating index (DI) was determined by dividing the difference in exploration time between the novel and familiar objects by the total exploration time. The combined time spent investigating both objects was also measured.

#### Forced Swimming Test (FST)

The forced swim test was performed according to established protocols [39, 40]. Prior to the test session, a pre-swim exposure was conducted 24 h earlier to screen the animals and exclude those exhibiting baseline depressive-like behavior, thereby ensuring a more uniform behavioral assessment during the test session. Each rat was individually placed in a transparent cylinder (45 cm in height, 20 cm in diameter) filled with about 35 cm of water maintained at 25 ± 1 °C for 5 min to establish an immobile posture on the test day. The immobility period was determined by measuring the time each animal remained immobile, defined as floating with minimal movements required to maintain the head above water.

### Biochemical Parameters

#### Assessment of Hepatotoxicity Markers

Serum AST and ALT levels were measured using a colorimetric assay; also serum albumin and total bilirubin were measured with commercially available kits, following the manufacturer’s protocols (Bio-Med Diagnostics Company, USA).

#### Evaluation of Oxidative Stress and Endogenous Antioxidant Markers

In hepatic tissue, lipid peroxidation was assayed via a TBARS kit, whereas the enzymatic activities of superoxide dismutase (SOD) and catalase (CAT) were assessed using corresponding commercial antioxidant kits, all executed in line with the manufacturer’s specifications (Bio-diagnostic, Egypt).

#### Enzyme-linked Immunosorbent Assay

Tissue samples were homogenized using phosphate-buffered saline (PBS; pH 7.4), centrifuged at 3000 rpm for 20 min, and the supernatant was collected for ELISA to measure IL-6, IL-1β, and TNF-α levels in rat colon tissue, using kits from R&D Systems, McKinley Place NE, Minneapolis, USA, Cat# R6000B, MyBioSource, San Diego, USA, Cat# MBS825017, and Cusabio, Houston, USA, Cat# CSB-E11987r, respectively. Anti-nuclear antibody (ANA) levels in rat liver tissue were measured using a kit from MyBioSource, San Diego, USA, Cat# MBS269217. Moreover, quinolinic acid and serotonin levels in rat hippocampal tissue were quantified using commercial assay kits from MyBioSource, San Diego, USA; Cat# MBS7269657 and MBS9362408, respectively. Every assay was executed in line with the guidelines provided by the manufacturers.

#### Western Blot Analysis

Protein expression profiling was executed by Western blot to evaluate Nrf2 and HO-1 in hepatic and hippocampal tissues, IDO in hippocampal tissue, and NF-κB in colonic tissue. Tissue homogenates were prepared using RIPA lysis buffer, which consists of 50 mM Tris-HCl, 150 mM NaCl, 0.1% SDS, 0.5% sodium deoxycholate, and 1% Triton X-100 at pH 8.0, supplemented with freshly prepared inhibitors targeting proteases and phosphatases. Protein content was measured using the Bradford Protein Assay Kit (Bio BASIC Inc., ON, Canada). Equalized protein aliquots (10 µg) were mixed with Laemmli buffer, denatured at 95 °C for 5 min, resolved by sodium dodecyl sulfate-polyacrylamide gel electrophoresis (SDS-PAGE) on 10% acrylamide gels, and transferred to PVDF membranes. Membranes were blocked with 5% bovine serum albumin (BSA) and incubated overnight at 4 °C with target-specific primary antibodies (Thermo Fisher Scientific, MA, USA): Nrf2 (Cat# PA5-105664), HO-1 (Cat# PA5-77833), IDO (Cat# PA5-79437), NF-κB (Cat# PA5-17150), and β-actin (Cat# PA1-183) were all employed at a dilution of 1:1000. Following the final wash steps, the membranes were treated with horseradish peroxidase (HRP)–tagged secondary antibodies for a duration of 2 h at 25 °C. Immunoreactive bands were revealed by an enhanced chemiluminescence (ECL) detection system, subjected to densitometric quantification with the ChemiDoc™ MP Imaging System (Bio-Rad, CA, USA), normalized to β-actin expression, and reported as arbitrary densitometric units. The uncropped original Western blot images are provided as Supplementary Material.

#### Histopathological and Immunohistochemical Examinations

After fixation in formalin (10%, neutral pH) for 72 h, liver, colon, and brain samples underwent dehydration in graded ethanol, clearing in xylene, and infiltration and embedding in Paraplast tissue embedding medium. Sections of 5-µm thickness were cut using a rotatory microtome to show hepatic parenchyma in various samples, intestinal wall, and sagittal brain sections to show hippocampus areas in various brain samples. The sections were thereafter affixed to glass slides, and tissue slices were stained with Hematoxylin and Eosin as a standard histological protocol. To demonstrate goblet cells and acidic mucins in a colon sample, alcian blue pH 2.5 was used, while brain samples were stained with toluidine blue stain to quantify intact neurons. Experienced histologists examined the samples blindly under a light microscope. All conventional sample fixation and staining protocols were carried out following accepted methodological guidelines [41].

A 5-micron-thick paraffin-embedded tissue section was produced for immunohistochemistry (IHC) examination. Immunohistochemistry was performed according to the manufacturer’s instructions. Depraffinized tissue sections were treated with 0.3% H_2_O_2_ for 20 min. The cells were then treated with anti-TLR4 antibody (bs-1021R - Bioss USA - 1:200), anti-ZO-1(1:1000 - Abcam - EPR19945-224), and anti-MMP-9 (GTX100458 - GeneTex Inc. - 1:200) overnight at 4 °C. Tissue slices were washed in PBS before being incubated with the secondary antibody HRP Envision kit (DAKO) for 20 min. They were then rinsed and incubated with diaminobenzidine (DAB) for 15 min. PBS washed, then hematoxylin was counterstained, dehydrated, and cleared in xylene before being cover slipped for microscopic analysis.

For histological evaluation of the brain, sections were analyzed as described by Abbas et al. [42], Specifically, six randomly chosen, non-overlapping fields from the CA3 region in each specimen were examined to determine the mean count of intact neurons in toluidine blue–stained sections, as well as the mean relative area percentage of neuronal MMP-9 expression in the hippocampus. Regarding liver specimens, and in line with Elsayed et al. [43], at least six randomly selected, non-overlapping microscopic fields from each liver section were analyzed to determine the mean relative area percentage of hepatocellular TLR4 immunohistochemical expression in the stained sections. Following the protocol described by Khedr et al. [44] at least six non-overlapping fields from each colonic specimen were randomly chosen and assessed by a histologist to measure the mucosal area percentage of reactive goblet cell mucin content, mean goblet cell diameter in alcian blue–stained sections, and the mean relative area percentages of TLR4 and ZO-1 expression in immunostained sections. All light microscopic assessments and image analyses were performed using the Leica Application Module for histological analysis integrated with a Full HD digital microscopic imaging system (Leica Microsystems GmbH, Germany).

### Statistical Evaluation

Statistical analysis was performed using GraphPad Prism version 9.3.1 Demo (GraphPad Software, San Diego, CA, USA). Data normality was assessed using the Shapiro-Wilk test prior to statistical analysis. The values were systematically assessed utilizing one-way ANOVA with Tukey’s post hoc comparison. The significance of differences in the duration of exploration of familiar and novel objects for each group was evaluated using two-way ANOVA, with objects and drug treatments designated as set variables. The values are presented as mean ± SD; a significance threshold was established at *P* < 0.05.

## Results

### AVA Alleviated LPS-induced Behavioral Changes in Rat

**Fig. 1:**
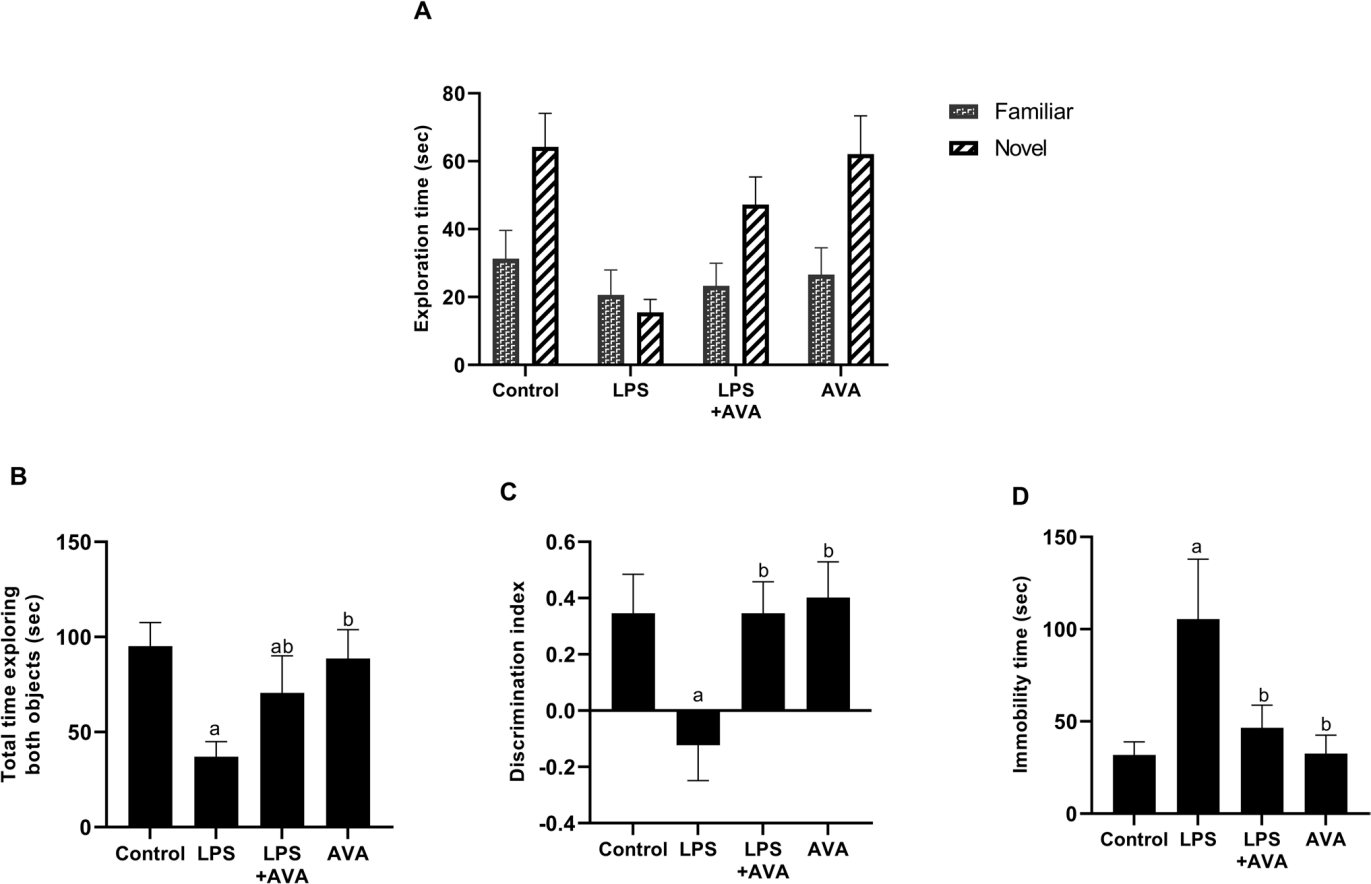
AVA alleviated behavioral changes in rats. **A** Exploration time of familiar and novel objects, **B** total time exploring both objects, **C** discrimination index measured during the novel object recognition assessment, and **D** immobility time in forced swimming test. The data (mean ± SD) underwent statistical evaluation utilizing one-way ANOVA with Tukey’s post hoc comparison. Exploration time differences for familiar and novel objects in each group were analyzed using two-way ANOVA. Statistical significance was indicated by “a” and “b,” representing differences from the normal control and LPS groups, respectively, at *P* < 0.05. LPS, lipopolysaccharide; AVA, avanafil

In the novel object identification test, rats treated with LPS showed no statistically significant difference in time spent examining the novel versus the familiar object. Furthermore, they demonstrated a statistically significant decline in time spent studying the novel object compared to the control group (75.86%). In addition, there was a significant decline in the discriminating index (135.39%) and total time spent studying both items (61.13%), which clearly indicated cognitive impairment. In contrast, rats treated with AVA demonstrated a significant elevation in the time spent exploring the novel object compared to the familiar one, with a twofold increase. They also spent threefold more time exploring the novel object than the LPS-treated group. Furthermore, the AVA-treated group showed a significant improvement in the discrimination index (382.14%) and a 1.9-fold increase in the total time spent exploring both objects compared to LPS group (Fig. 1A–C).

Figure 1D depicts that treatment of animals with LPS in the forced swimming test significantly increased immobility time by 231.45%, when compared with control group. On the other hand, immobility time was significantly reduced by 55.88% in animals treated with AVA after LPS administration as compared to LPS alone group.

### AVA Enhances Intestinal Barrier Integrity Via Upregulation of ZO-1 Expression in Rat Colon

**Fig. 2:**
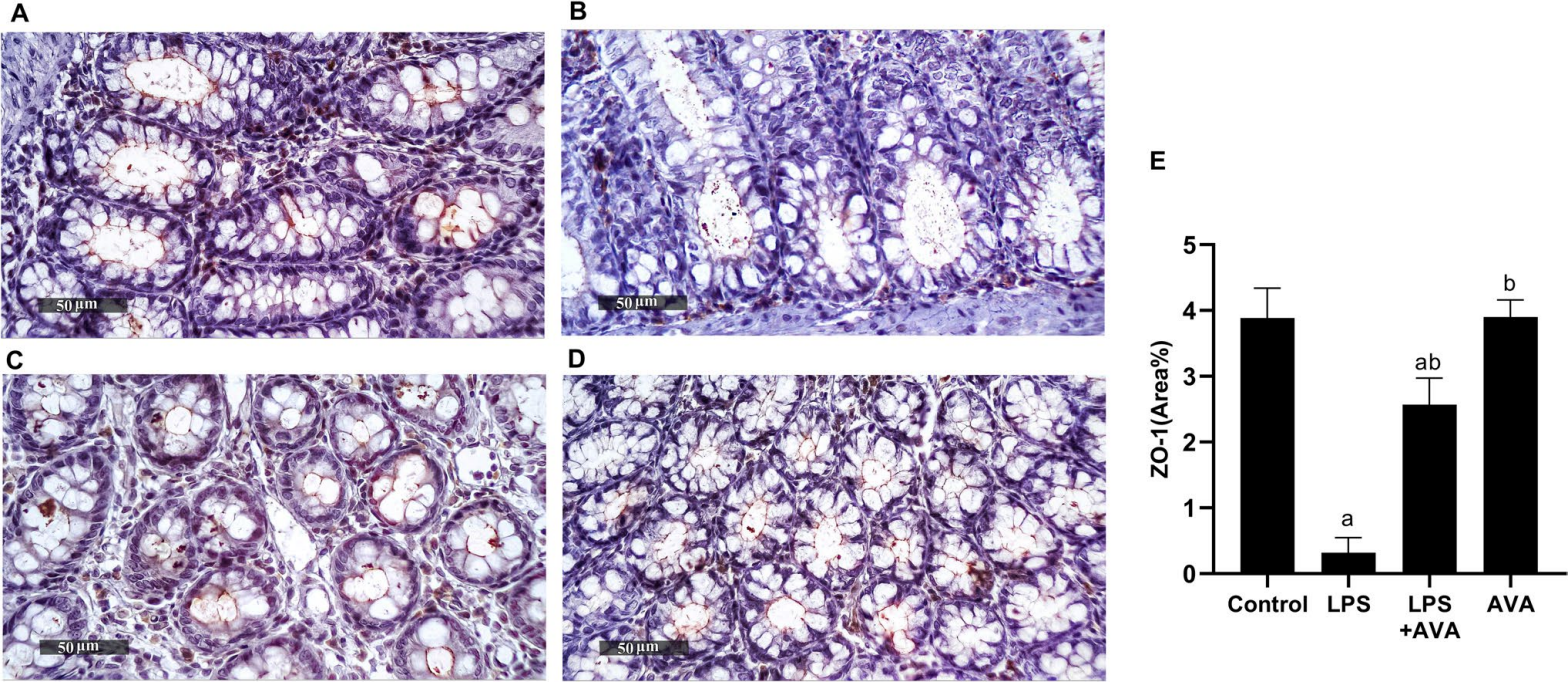
AVA reduces the expression of ZO-1 within the rat colon. Photomicrographs depicting immunohistochemical staining of ZO-1 in the rat colon. **A** Control group, **B** LPS group, **C** LPS+AVA-treated group, **D** AVA alone treatment, and **E** quantification of ZO-1 area percentage. The data (mean ± SD) underwent statistical evaluation utilizing one-way ANOVA with Tukey’s post hoc comparison. Statistical significance was indicated by “a” and “b,” representing differences from the normal control and LPS groups, respectively, at *P* < 0.05. LPS, lipopolysaccharide; AVA, avanafil; ZO-1, Zonula Occludens-1

The LPS challenge decreased the abundances of ZO-1 in colon by 91.85%, relative to the control group. Oral AVA increased the protein level of ZO-1 in colon by 710.53%, compared to the rats challenged with LPS (Fig. 2).

### AVA Attenuates LPS-induced Inflammatory Cascade in Rat Colon

**Fig. 3:**
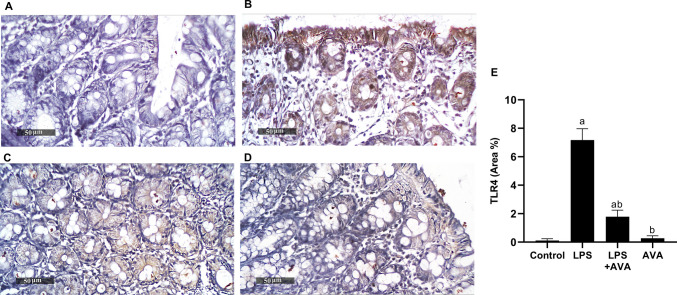
AVA suppresses TLR4 expression in rat colon. photomicrographs depicting immunohistochemical staining of TLR4 in rat colon. **A** Control group, **B** LPS group, **C** LPS+AVA-treated group, **D** AVA alone treatment, and **E** quantification of TLR4 area percentage. The data (mean ± SD) underwent statistical evaluation utilizing one-way ANOVA with Tukey’s post hoc comparison. Statistical significance was indicated by “a” and “b,” representing differences from the normal control and LPS groups, respectively, at *P* < 0.05. LPS, lipopolysaccharide; AVA, avanafil; TLR4, Toll-like receptor 4

Rats treated solely with LPS displayed a substantial 6042.86% increase in TLR4 expression. However, when compared to rats treated with LPS alone, AVA dramatically reduced the expression of TLR4 triggered by LPS in rat colon by 75.12% (Fig. 3).

**Fig. 4:**
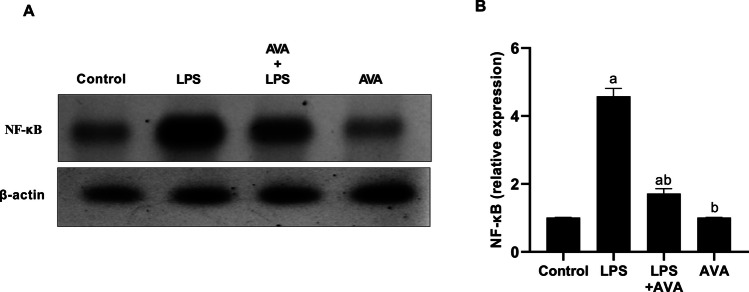
AVA downregulates NF-κB expression in rat colon. **A** NF-κB and β-actin were detected in total extracts from the rat colon using Western blot analysis and **B** densitometric measurement of NF-κB was carried out in relation to β-actin expression. The data (mean ± SD) underwent statistical evaluation utilizing one-way ANOVA with Tukey’s post hoc comparison. Statistical significance was indicated by “a” and “b,” representing differences from the normal control and LPS groups, respectively, at *P* < 0.05. LPS, lipopolysaccharide; AVA, avanafil; NF-κB, nuclear factor-kappa B

NF-κB expression was significantly elevated in rat colon given LPS by 353.80%. However, compared to rats treated with LPS alone, animals treated with AVA showed significantly decreased levels of NF-κB expression by 62.55% (Fig. 4).

**Fig. 5:**
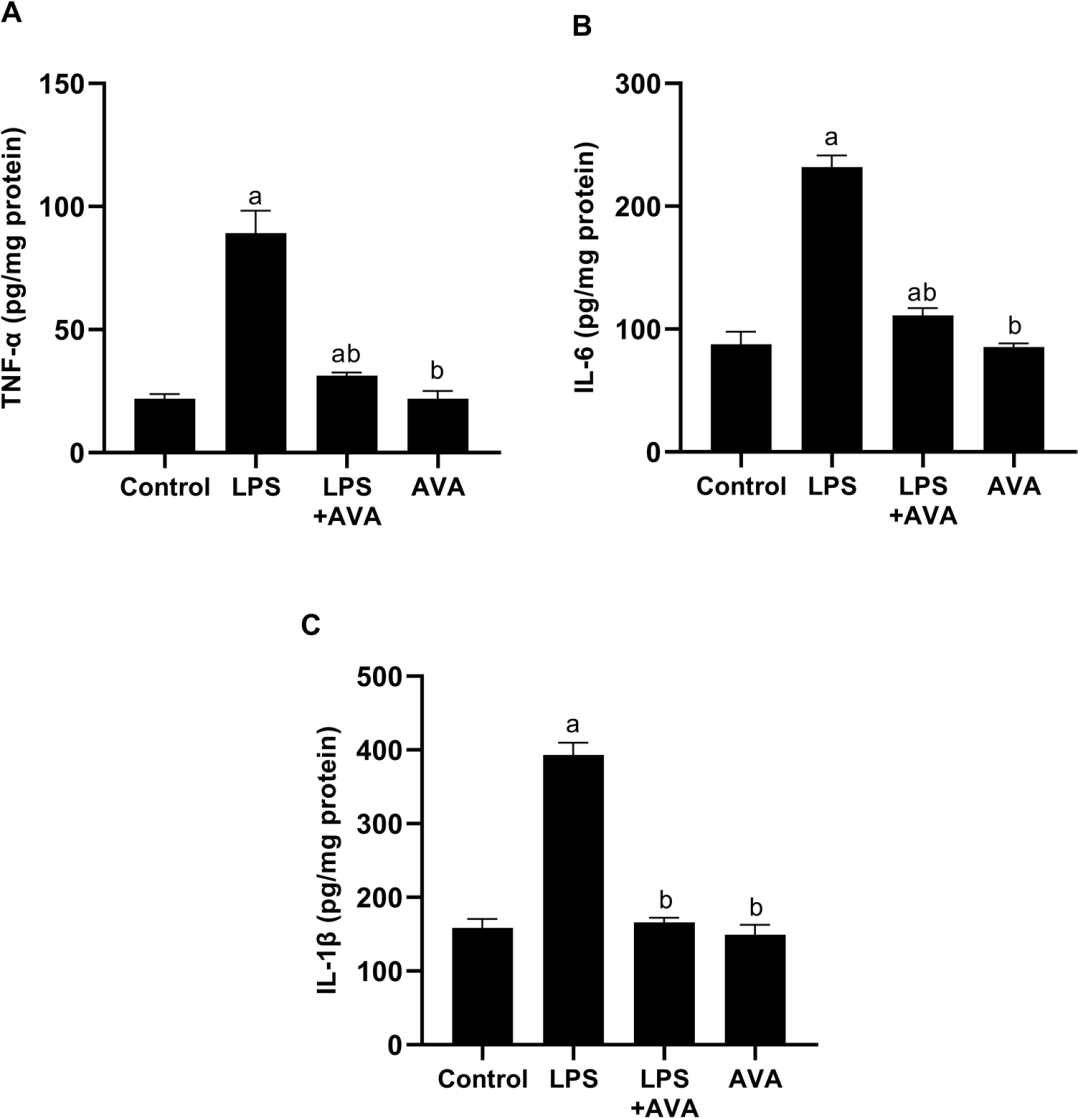
AVA suppress pro-inflammatory cytokines levels in rat colon. **A** TNF-α, **B** IL-6, and **C** IL-1β. The data (mean ± SD) underwent statistical evaluation utilizing one-way ANOVA with Tukey’s post hoc comparison. Statistical significance was indicated by “a” and “b,” representing differences from the normal control and LPS groups, respectively, at *P* < 0.05. TNF-α, tumor necrosis factor-α; IL-6, interleukin-6; LPS, lipopolysaccharide; AVA, avanafil; IL-1β, interleukin-1β

Evaluation of pro-inflammatory cytokines TNF-α, IL-6, and IL-1β demonstrated that LPS administration induced significant increases of 307.38%, 164.47%, and 147.84%, respectively, versus controls. AVA therapy, in contrast, resulted in significant reductions of 64.95%, 52.06%, and 57.74% in pro-inflammatory cytokines, respectively, in comparison to LPS-exposed animals (Fig. 5A–C).

### AVA Improves Histopathological Changes Induced by LPS in Rat Colon

**Fig. 6:**
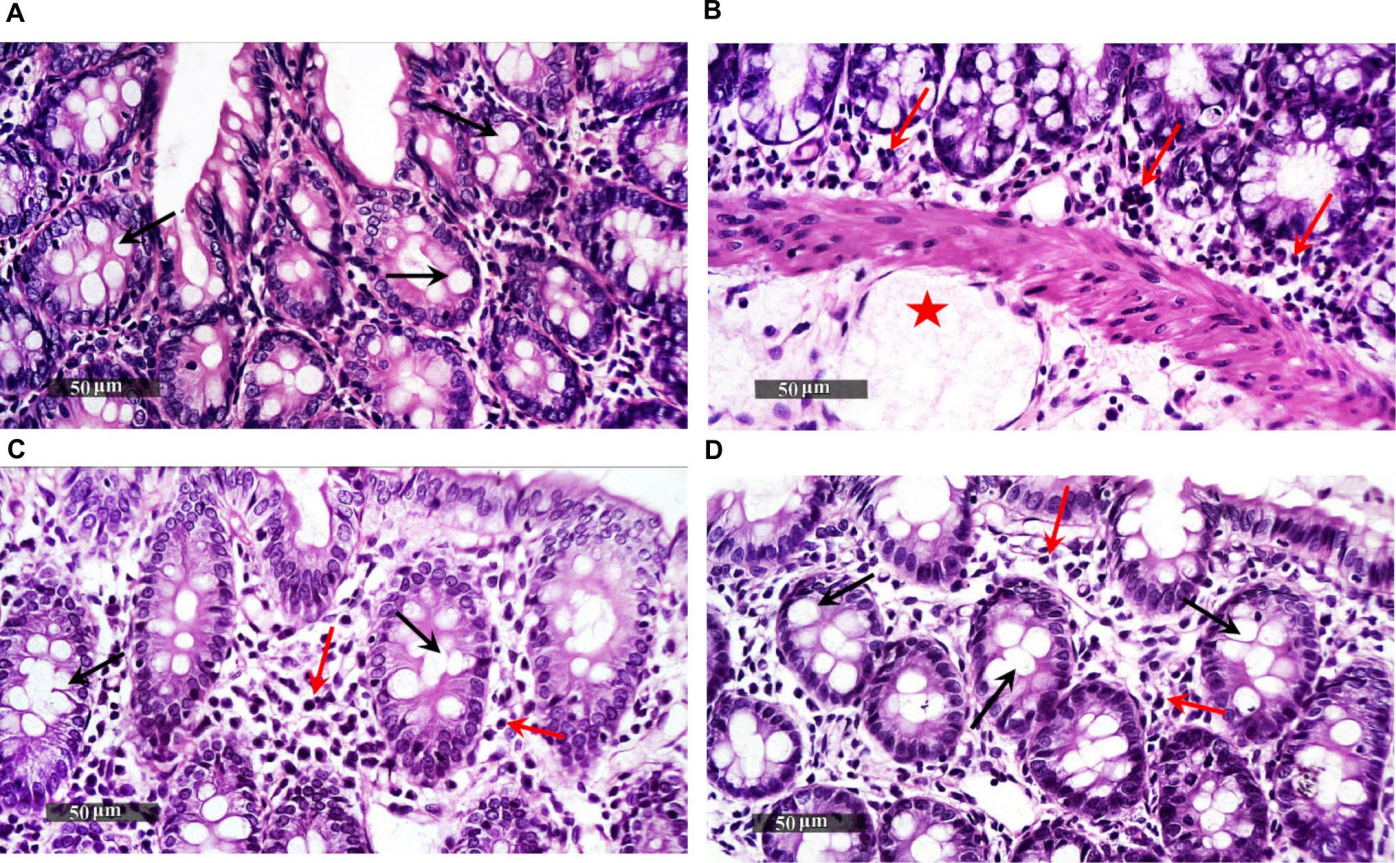
AVA improves histopathological changes in rat colon. Microscopic views of colon sections processed with hematoxylin and eosin staining and examined at ×200 magnification. **A** Section derived from control group depicting normal organized morphological features of colon wall with intact intestinal crypts displayed abundant goblet cells (black arrow). **B** Section derived from LPS-treated group depicting remarkable mucosal disorganization and loss of morphological features of colonic wall including loss of glandular goblet cells densities accompanied moderate to severe inter glandular inflammatory cells infiltrates (red arrow) with submucosal-edema and dilated lymphatic vessels (red star). **C** Section derived from a rat treated with LPS+AVA depicting significant improvement of colonic wall glandular structures with abundant figures of apparent intact lining epithelium and mucosal glandular elements with a significant enhanced mature goblet cells maturity and differentiation (black arrow) and moderate persistent of subepithelial inflammatory cells were observed in most of examined samples (red arrow). **D** Sections derived from AVA group depicting apparent intact intestinal crypts displayed abundant goblet cells, resembling normal controls. LPS, lipopolysaccharide; AVA, avanafil

Colon sections from the control group showed normal arranged morphological characteristics of the colon wall, including intact intestinal crypts with plenty of goblet cells, intact covering epithelium with a normal submucosa, and an outside muscular coat (Fig. 6A), while those of LPS group showed remarkable mucosal disorganization and loss of morphological features of colonic wall including loss of glandular goblet cells densities accompanied by moderate to severe inter glandular inflammatory cells infiltrates with submucosal edema and dilated lymphatic vessels (Fig. 6B). Colon sections of LPS+AVA group showed significant improvement of colonic wall glandular structures with abundant figures of apparent intact lining epithelium and mucosal glandular elements with a significant enhanced mature goblet cells maturity and differentiation and moderate persistence of subepithelial inflammatory cells were observed in most of examined samples (Fig. 6C). Colon specimens of rats treated with AVA alone demonstrated preserved intestinal crypt integrity and abundant goblet cells, resembling the histological features of normal controls (Fig. 6D).

**Fig. 7:**
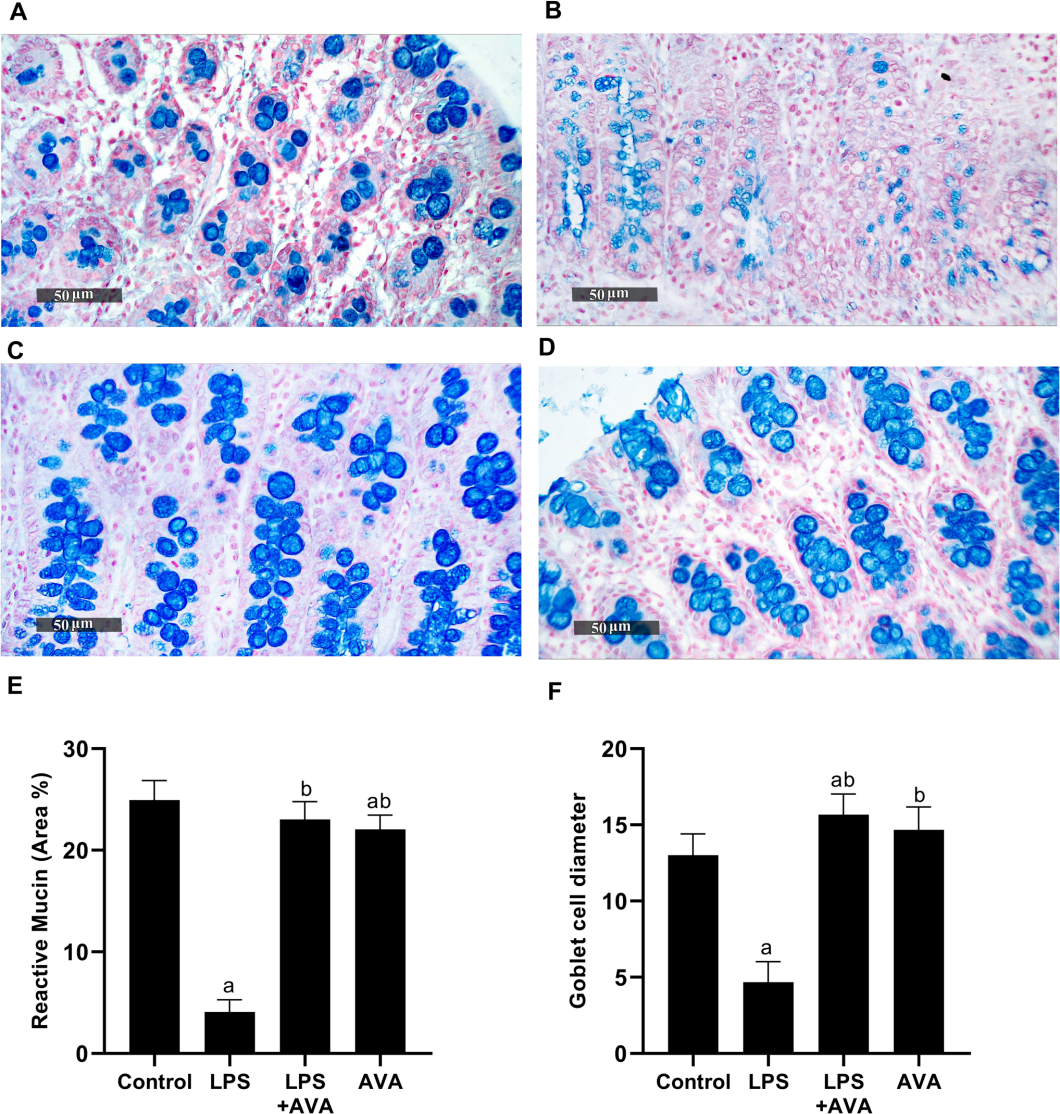
AVA restores goblet cell integrity and mucin content in LPS-treated rat colon shown by alcian blue staining. **A** Control group, **B** LPS group, **C** LPS+AVA-treated group, **D** AVA alone treatment, **E** quantification of reactive mucin area percentage, and **F** quantification of goblet cell diameter. The data (mean ± SD) underwent statistical evaluation utilizing one-way ANOVA with Tukey’s post hoc comparison. Statistical significance was indicated by “a” and “b,” representing differences from the normal control and LPS groups, respectively, at *P* < 0.05. LPS, lipopolysaccharide; AVA, avanafil

In addition, alcian blue staining was applied to assess histological changes in the colon of the rats. The results showed that, compared with the standard group, goblet cell diameter in the LPS group was significantly reduced by 64.10%, whereas AVA treatment markedly increased by 235.71%. Similarly, the percentage of reactive mucin decreased by 83.62% in the LPS group but was substantially restored by AVA treatment, showing an increase of 464.08%, as displayed in Fig. 7.

### AVA Suppresses IDO Expressions in the Rat Hippocampus

**Fig. 8:**
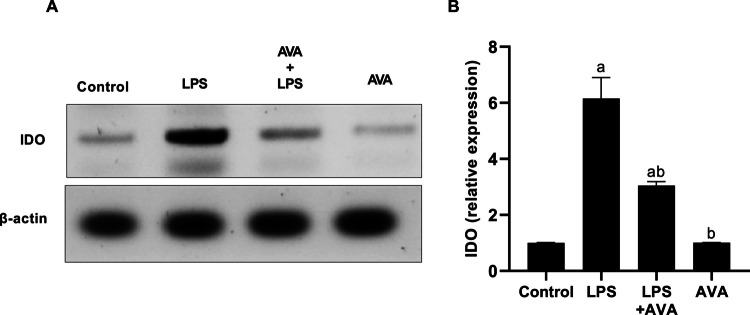
AVA downregulates IDO expression in rat hippocampus. **A** IDO and β-actin were detected in total extracts from the rat hippocampus using Western blot analysis and **B** densitometric measurement of IDO was carried out in relation to β-actin expression. The data (mean ± SD) underwent statistical evaluation utilizing one-way ANOVA with Tukey’s post hoc comparison. Statistical significance was indicated by “a” and “b,” representing differences from the normal control and LPS groups, respectively, at *P* < 0.05. LPS, lipopolysaccharide; AVA, avanafil; IDO, indoleamine 2,3-dioxygenase

Numerous lines of evidence suggest that stress-induced neuroinflammation can activate the IDO enzyme, which aids in the development of depression [45]. We assessed IDO expressions in the rat hippocampus to evaluate the kynurenine pathway’s activity. In comparison to the control group, animals treated with LPS exhibited a significant (511.42%) increase in IDO enzyme expression (Fig. 8). However, compared to animals treated with LPS alone, IDO expression dramatically dropped by 50.45% in animals treated with AVA following LPS injection.

### IDO Inhibition Restores Kynurenine Pathway Balance and Serotonin Levels

**Fig. 9:**
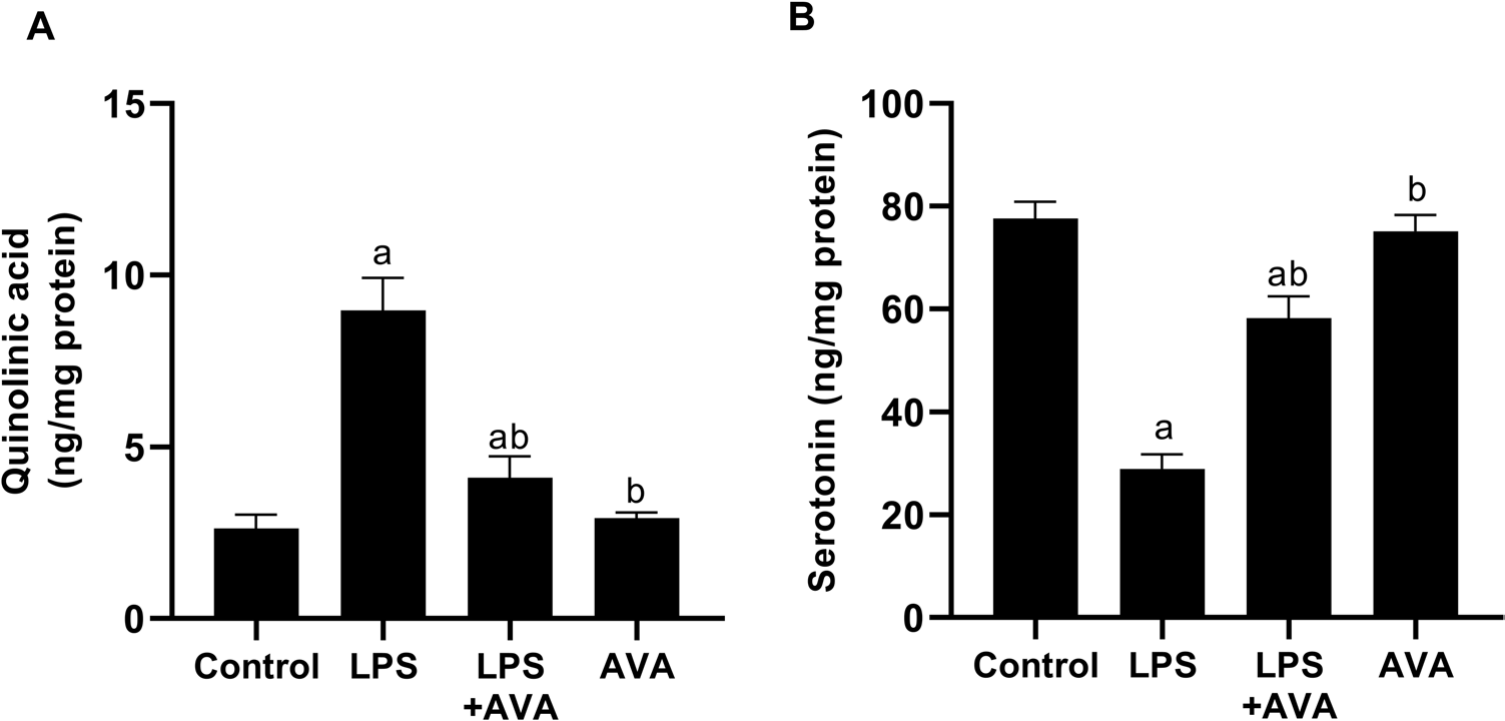
AVA downregulates quinolinic acid and restores serotonin level in rat hippocampus. **A** Quinolinic acid and **B** serotonin. The data (mean ± SD) underwent statistical evaluation utilizing one-way ANOVA with Tukey’s post hoc comparison. Statistical significance was indicated by “a” and “b,” representing differences from the normal control and LPS groups, respectively, at *P* < 0.05. LPS, lipopolysaccharide; AVA, avanafil

As illustrated in Fig. 9, serotonin and its metabolites were assessed in the hippocampi of rats retrieved at the termination of the experiment to elucidate the mechanism by which alterations in tryptophan metabolism contribute to MDD. LPS injection promoted the shunting of tryptophan metabolism toward quinolinic acid rather than serotonin, under the influence of inflammation mediated by LPS-induced activation of IDO. This resulted in a significant increase in quinolinic acid by 240.72% and a decrease in serotonin level by 62.75%, compared to the normal control group. However, treatment with AVA reversed this effect, leading to a reduction in tryptophan breakdown into quinolinic acid by 54.28% and restoration of serotonin levels in rat hippocampus by 101.21% relative to the LPS group.

### AVA Suppresses MMP-9 Expression in the Rat Hippocampus

**Fig. 10:**
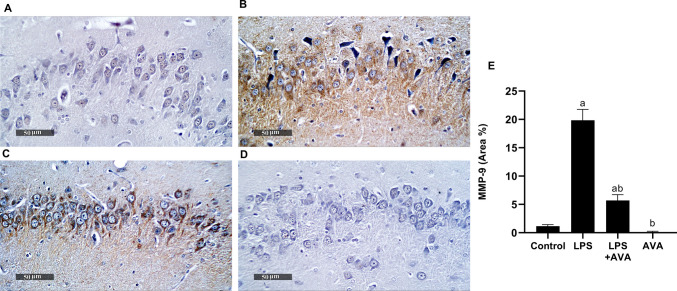
AVA downregulates hippocampal expression of MMP-9. Photomicrographs showing immunohistochemical staining of MMP-9 in rat hippocampus. **A** Control group, **B** LPS group, **C** LPS+AVA-treated group, **D** AVA alone treatment, and **E** quantification of MMP-9 area percentage. The data (mean ± SD) underwent statistical evaluation utilizing one-way ANOVA with Tukey’s post hoc comparison. Statistical significance was indicated by “a” and “b,” representing differences from the normal control and LPS groups, respectively, at *P* < 0.05. LPS, lipopolysaccharide; AVA, avanafil; MMP-9, matrix metalloproteinase-9

Animals receiving LPS alone exhibited a marked (1624.64%) elevation in MMP-9 expression relative to the normal control group. In contrast, AVA treatment markedly attenuated the LPS-induced upregulation of MMP-9 in the rat hippocampus by 71.26% compared with the LPS-only group (Fig. 10).

### AVA Potentiates the Expression of Nrf2 and HO-1 in Rat Hippocampal Tissue

**Fig. 11:**
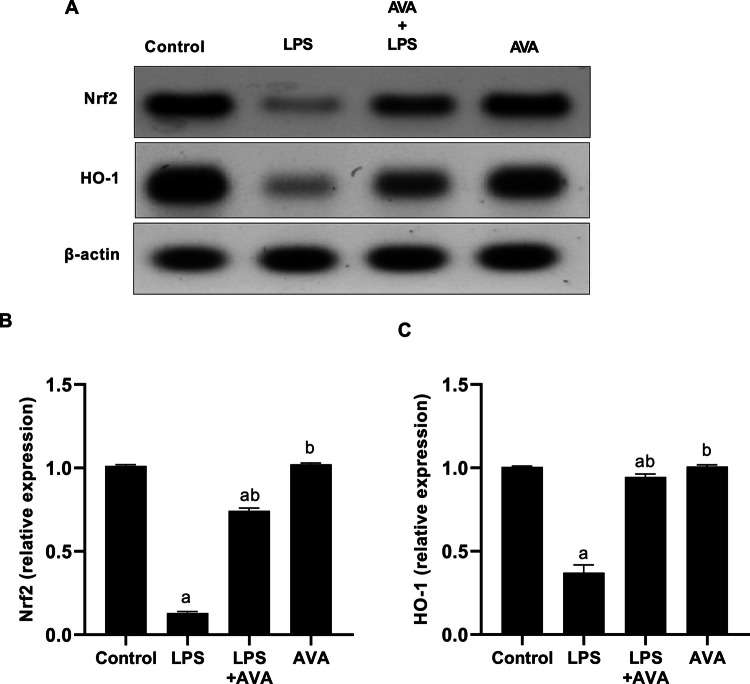
AVA increase Nrf2 and HO-1 expression in rat hippocampus. **A** Nrf2, HO-1, and β-actin were detected in total extracts from the rat hippocampus using Western blot analysis, **B** densitometric measurement of Nrf2 was carried out in relation to β-actin expression, and **C** densitometric measurement of HO-1 was carried out in relation to β-actin expression. The data (mean ± SD) underwent statistical evaluation utilizing one-way ANOVA with Tukey’s post hoc comparison. Statistical significance was indicated by “a” and “b,” representing differences from the normal control and LPS groups, respectively, at *P* < 0.05. LPS, lipopolysaccharide; AVA, avanafil; Nrf2, nuclear factor erythroid 2–related factor 2; HO-1, heme oxygenase-1

As seen in Fig. 11, in comparison to the normal control group, LPS injection reduced Nrf2 expression in the rat hippocampus by 87.15%. In contrast to the group treated with LPS alone, rats treated with AVA had a 471.79% increase in Nrf2 expression. Expression of HO-1 enzyme, which is downstream of the Nrf2 signaling pathway, was similarly (63.02%) lower in the LPS group. However, when compared to rats challenged with LPS alone, animals treated with AVA depicted a significant rise in HO-1 expression by 154.26%.

### AVA Ameliorates LPS-induced Histopathological Alterations in the Rat Hippocampus

**Fig. 12:**
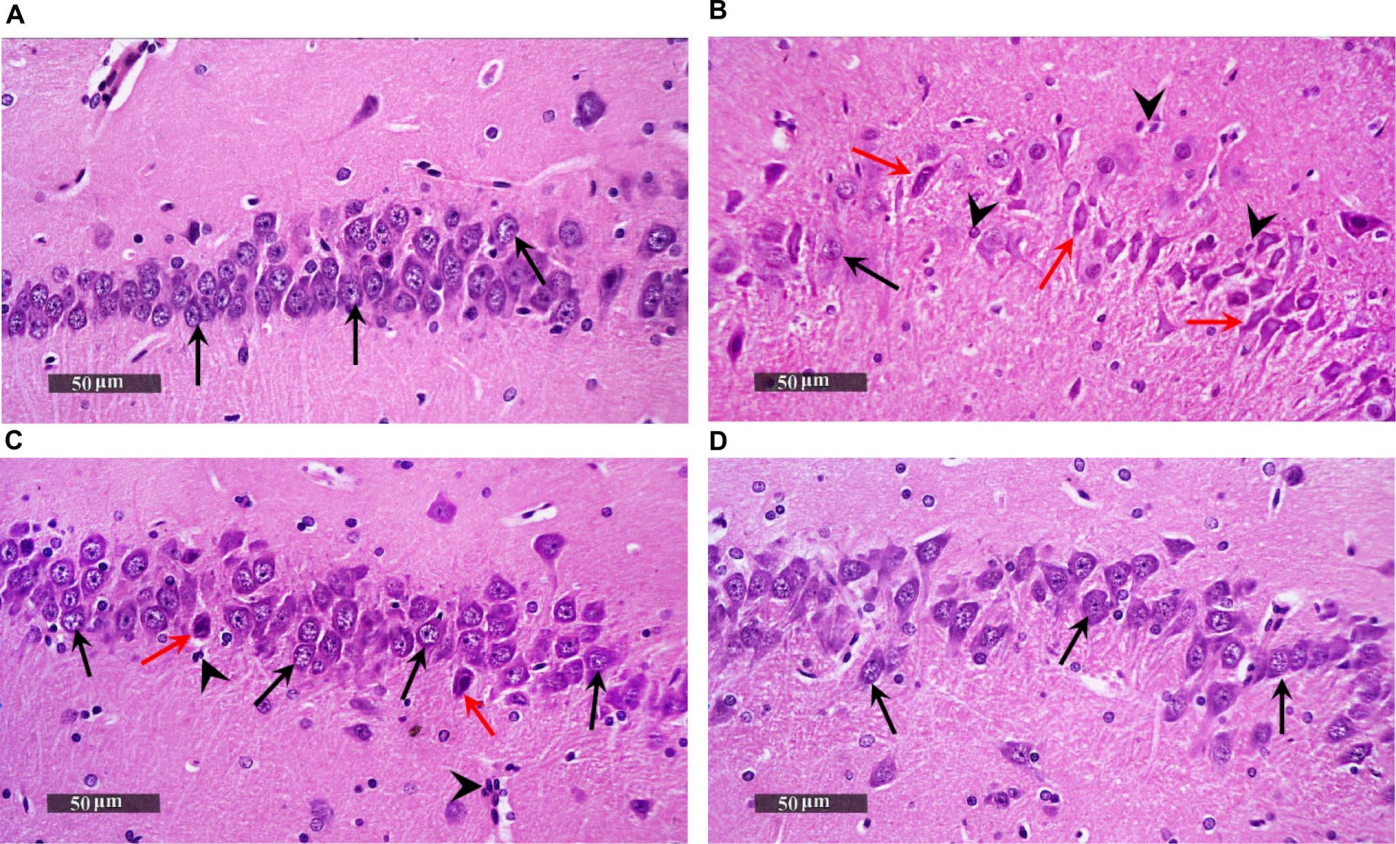
AVA improves hippocampal histopathological features. Microscopic views of hippocampus sections processed with hematoxylin and eosin staining and examined at ×200 magnification. **A** Section obtained from control group displayed well-preserved histological architecture of the hippocampal layers, characterized by an intact, sharply demarcated pyramidal neuronal layer containing numerous vesicular nuclei with distinct subcellular detail (black arrows). The intercellular brain matrix appeared structurally preserved, with no histological evidence of degenerative alterations. **B** Section obtained from LPS-treated group showing significant neurodegeneration was observed within the CA3 pyramidal neuron population with marked distorted and lacked normal structure and presented pyknotic nuclei and grossly disturbed subcellular features (red arrow), alternating with rare visible intact neurons (black arrow). Changes were accompanied by moderate perineuronal edema and a moderate increase in reactive glial cells. This included hypertrophic astrocytes and activated microglia (arrowhead). (**C**) Section obtained from a rat treated with LPS+AVA showing presented evident neuroprotective efficacy with remarkable higher figures of apparent intact neurons (black arrow), sporadic figures of neuronal degenerative changes (red arrow). Moreover, persistent moderate glial cells infiltrates were shown (arrowhead). **D** Sections obtained from AVA group showing the same histological organization as normal control samples. LPS, lipopolysaccharide; AVA, avanafil

Hippocampal CA3 sections of control group revealed consistently conserved structural organization within the hippocampal layers; this architecture featured a structurally intact, distinctly bound pyramidal neuronal layer containing multiple clear nuclei with conspicuous nucleoli and cytoplasmic detail. The intercellular brain matrix appeared structurally preserved, with no histological evidence of degenerative alterations (Fig. 12A). In contrast, the LPS-treated group exhibited pronounced neurodegeneration within the CA3 pyramidal neuron layer, characterized by severe distortion and loss of normal cellular architecture. Neurons displayed pyknotic nuclei and markedly disrupted subcellular structures, with only a few neurons retaining an apparently intact morphology. These degenerative changes were accompanied by moderate perineuronal edema and a noticeable increase in reactive glial cells, including hypertrophic astrocytes and activated microglia (Fig. 12B). Hippocampal CA3 sections of LPS+AVA group presented evident neuroprotective efficacy with remarkable higher figures of apparent intact neurons and sporadic figures of neuronal degenerative changes. Moreover, persistent moderate glial cells infiltrates (Fig. 12C). Hippocampal CA3 sections of rats treated with AVA alone showed the same histological organization as normal control samples without abnormal alteration records (Fig. 12D).

**Fig. 13:**
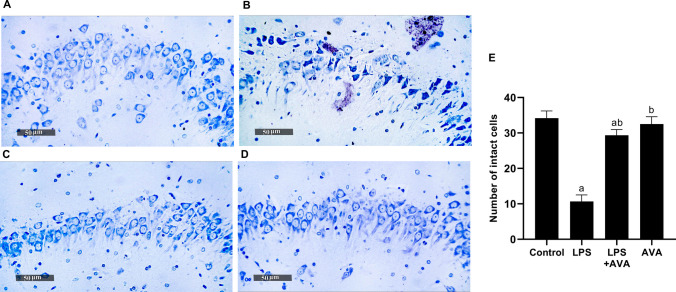
AVA attenuates neuronal damage in the hippocampal CA3 region as shown by toluidine blue staining. **A** Control group, **B** LPS group, **C** LPS+AVA-treated group, **D** AVA alone treatment, and **E** quantification of number of intact cells. The data (mean ± SD) underwent statistical evaluation utilizing one-way ANOVA with Tukey’s post hoc comparison. Statistical significance was indicated by “a” and “b,” representing differences from the normal control and LPS groups, respectively, at *P* < 0.05. LPS, lipopolysaccharide; AVA, avanafil

In addition, toluidine blue was used to detect the histological changes in rat hippocampus. The results revealed that the number of intact cells in the hippocampus of the rats in the LPS group was significantly reduced by 68.78%, compared with the rat in the control group. This decrease was markedly upregulated by 175% after AVA treatment, compared to LPS group (Fig. 13).

### AVA Preserves Serum Markers of Liver Function

**Fig. 14:**
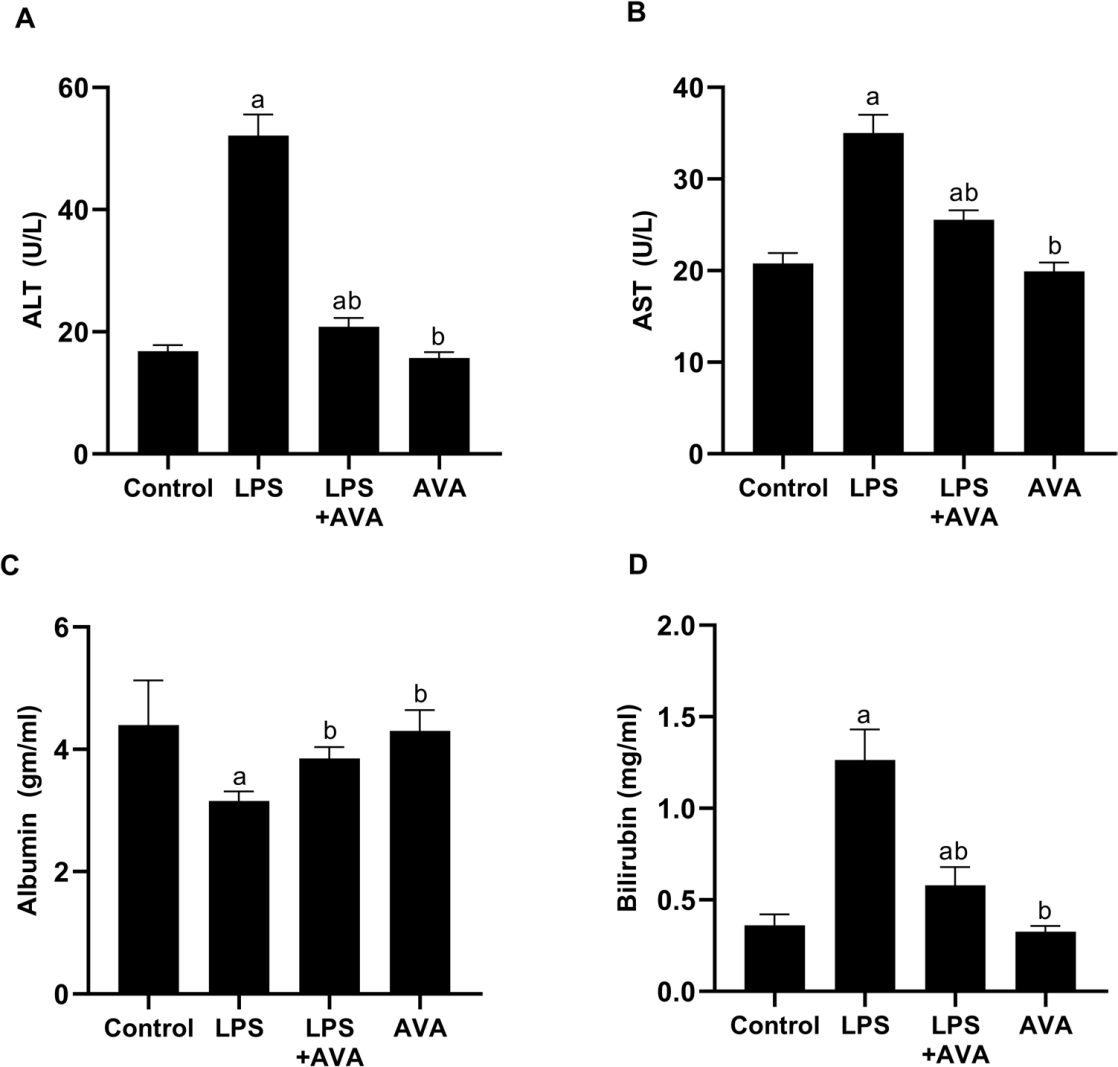
AVA upholds serum markers associated with liver function. ALT (**A**), AST (**B**), albumin (**C**), and bilirubin (**D**) levels in rats. The data (mean ± SD) underwent statistical evaluation utilizing one-way ANOVA with Tukey’s post hoc comparison. Statistical significance was indicated by “a” and “b,” representing differences from the normal control and LPS groups, respectively, at *P* < 0.05. LPS, lipopolysaccharide; AVA, avanafil; ALT, alanine aminotransferase; AST, aspartate aminotransferase

As depicted in Fig. 14A, B, and D, LPS treatment caused a significant increase in serum ALT and AST activities by 209.81% and 68.40%, respectively, versus controls. AVA administration, however, led to a marked reduction in ALT and AST activities by 60.08% and 27%, respectively, compared to the LPS group. Additionally, the administration of LPS dramatically raised bilirubin levels by 251.39% in comparison to the control group. However, compared to animals treated with LPS alone, animals treated with AVA had a substantial 54.15% reduction in bilirubin levels.

Additionally, a notable reduction (27.88%) in albumin levels was observed in animals challenged with LPS versus controls, suggesting diminished hepatic function. However, AVA administration considerably restored liver function, as reflected by a 20.27% elevation in albumin levels relative to the LPS-only group (Fig. 14C).

### AVA Reduces Hepatic Oxidative Damage Triggered by LPS in Rat Liver

**Fig. 15:**
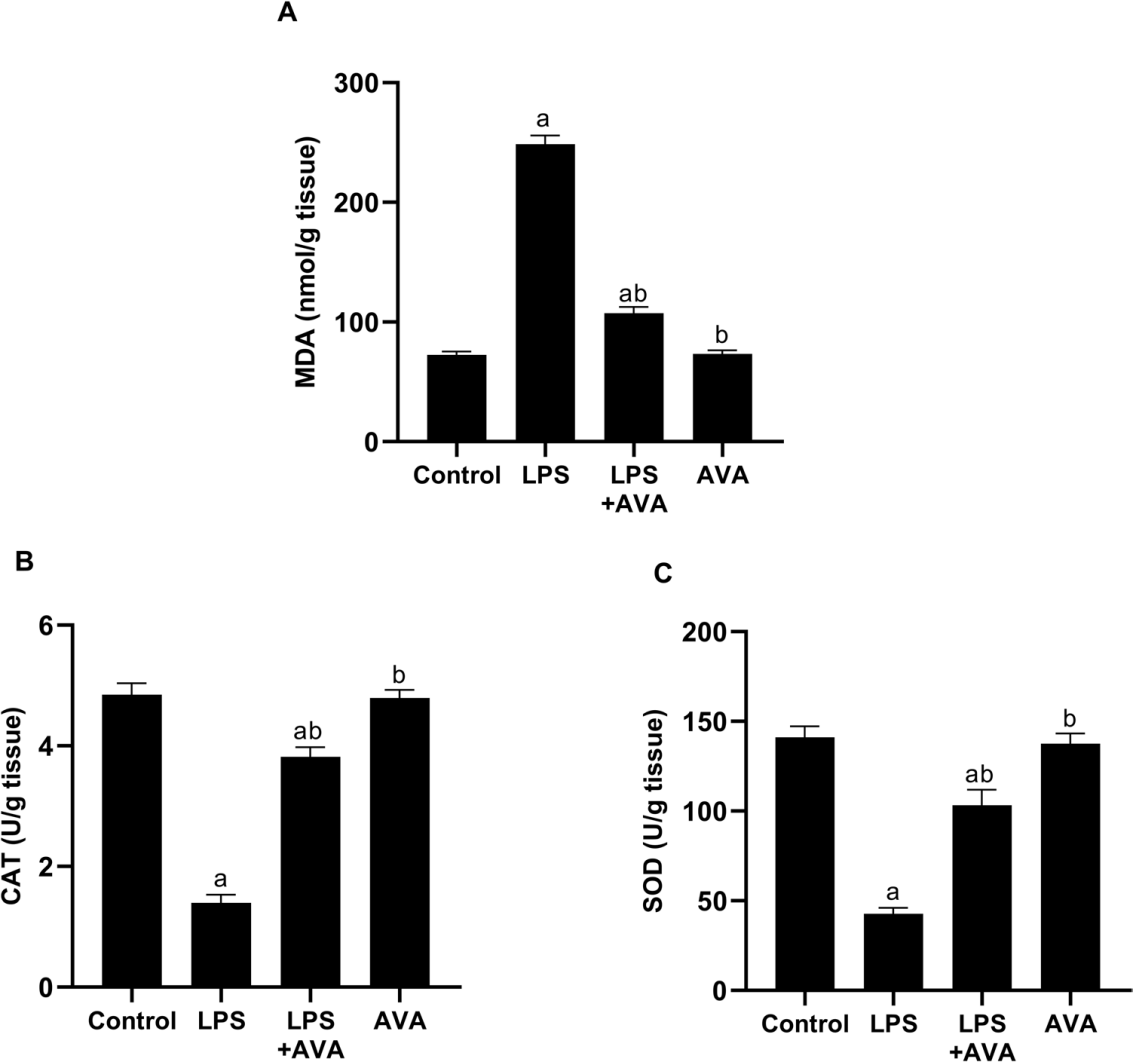
AVA ameliorates oxidative stress in hepatic tissue. **A** MDA, **B** CAT, and **C** SOD. The data (mean ± SD) underwent statistical evaluation utilizing one-way ANOVA with Tukey’s post hoc comparison. Statistical significance was indicated by “a” and “b,” representing differences from the normal control and LPS groups, respectively, at *P* < 0.05. LPS, lipopolysaccharide; AVA, avanafil; MDA, malondialdehyde; CAT, catalase; SOD, superoxide dismutase

The byproduct of lipid peroxidation (MDA) increased by 241.85% in the animals treated with LPS as compared to the normal control group, highlighting the oxidative damage in the liver attributable to LPS. However, compared to the LPS group, AVA therapy considerably reduced the amount of lipid peroxidation caused by LPS by 56.86% (Fig. 15A).

Furthermore, compared to the normal control group, the injection of LPS significantly reduced the hepatic activities of CAT and SOD by 71.21% and 69.76%, respectively. However, compared to rats treated with LPS alone, CAT and SOD activities were significantly elevated in animals treated with AVA by 173.73% and 141.94%, respectively (Fig. 15B and C).

### AVA Protects Against LPS-induced Hepatic Autoimmunity by Reducing ANA in Rat Liver

**Fig. 16:**
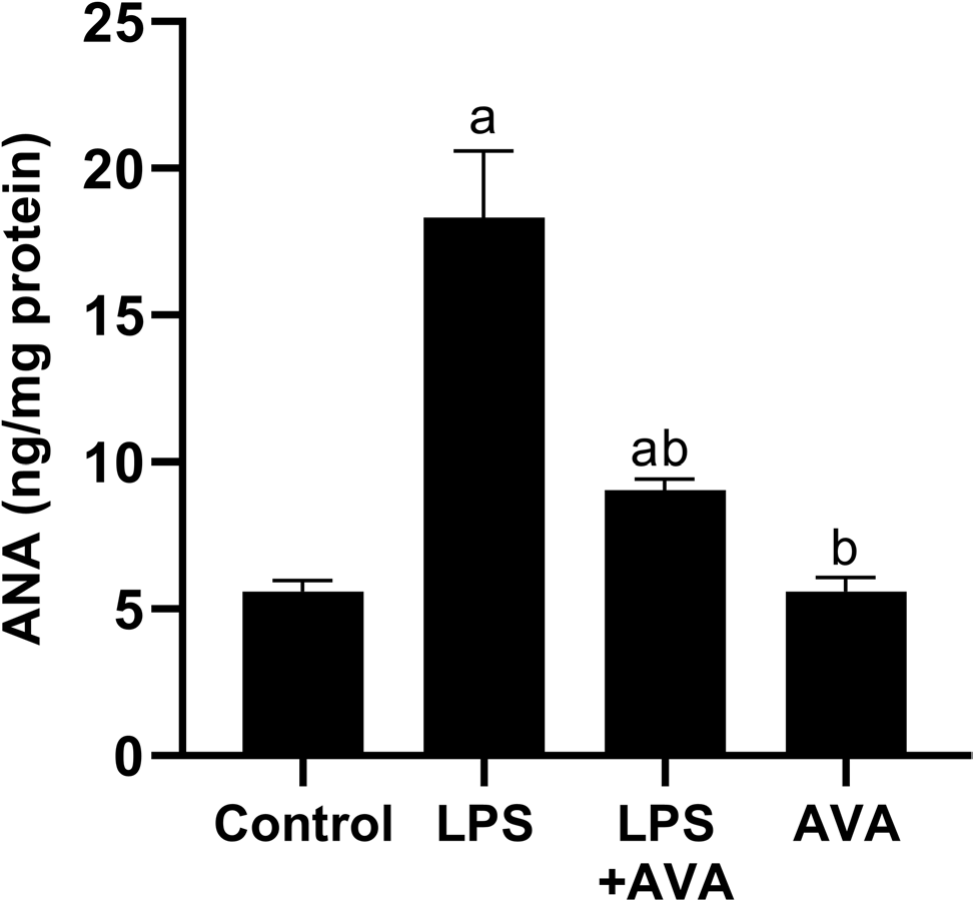
AVA reduces liver content of ANA. The data (mean ± SD) underwent statistical evaluation utilizing one-way ANOVA with Tukey’s post hoc comparison. Statistical significance was indicated by “a” and “b,” representing differences from the normal control and LPS groups, respectively, at *P* < 0.05. LPS, lipopolysaccharide; AVA, avanafil; ANA, anti-nuclear antibody

To assess AIH, ANA testing was performed across experimental groups. As demonstrated in Fig. 16, ANA expression was markedly elevated in LPS-treated rats, showing 227.97% increase compared with controls. Treatment with AVA significantly reduced ANA expression by 50.57% relative to the LPS group.

### AVA Downregulates TLR4 Induced by LPS in Rat Liver

**Fig. 17:**
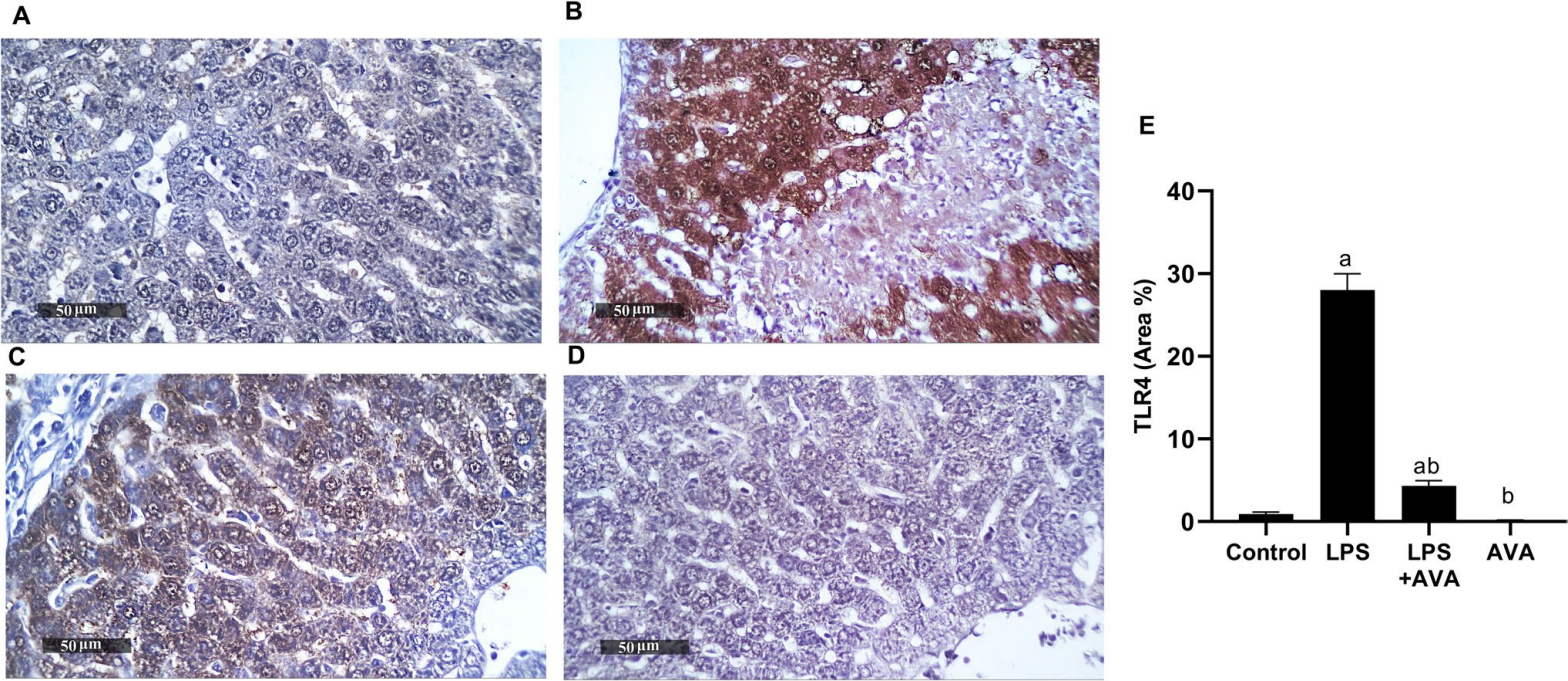
AVA attenuates hepatic TLR4 expression. photomicrographs depicting immunohistochemical staining of TLR4 in rat liver. **A** Control group, **B** LPS group, **C** LPS+AVA-treated group, **D** AVA alone treatment, and **E** quantification of TLR4 area percentage. The data (mean ± SD) underwent statistical evaluation utilizing one-way ANOVA with Tukey’s post hoc comparison. Statistical significance was indicated by “a” and “b,” representing differences from the normal control and LPS groups, respectively, at *P* < 0.05. LPS, lipopolysaccharide; AVA, avanafil; TLR4, Toll-like receptor 4

In comparison to the normal control group, rats treated with LPS alone showed a significant increase in TLR4 expression by 2956.36%. However, compared to rats treated with LPS alone, AVA considerably reduced LPS-induced TLR4 expression in rat liver by 84.59% (Fig. 17).

### AVA Potentiates Nrf2 and HO‑1 Signaling Pathway in Rat Liver

**Fig. 18:**
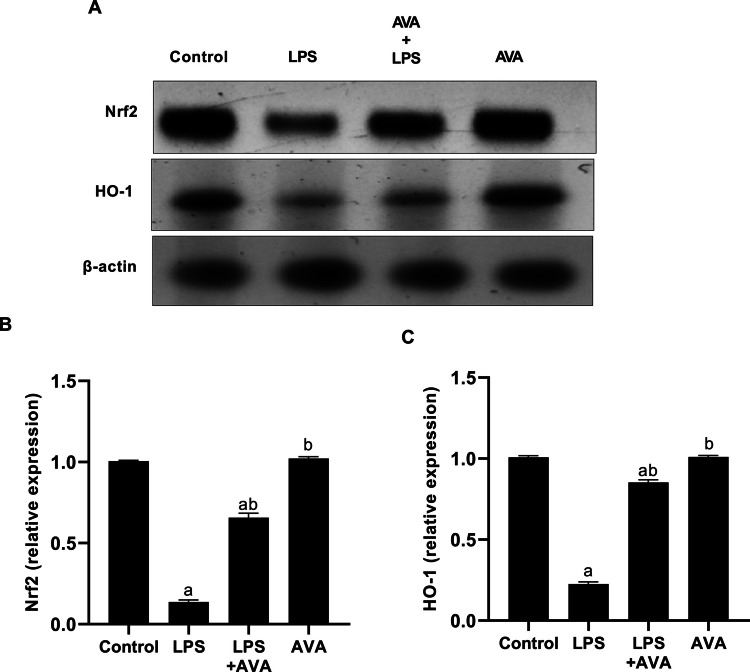
AVA suppresses Nrf2 and HO-1 expression in rat hepatic tissue. **A** Nrf2, HO-1, and β-actin were detected in total extracts from the rat liver using Western blot analysis, **B** densitometric measurement of Nrf2 was carried out in relation to β-actin expression, and **C** densitometric measurement of HO-1 was carried out in relation to β-actin expression. The data (mean ± SD) underwent statistical evaluation utilizing one-way ANOVA with Tukey’s post hoc comparison. Statistical significance was indicated by “a” and “b,” representing differences from the normal control and LPS groups, respectively, at *P* < 0.05. LPS, lipopolysaccharide; AVA, avanafil; Nrf2, nuclear factor erythroid 2–related factor 2; HO-1, heme oxygenase-1

As Fig. 18 illustrates, LPS injections reduced Nrf2 expression in rat liver by 86.40% when compared to the normal control group. In contrast to the group that received LPS alone, rats treated with AVA following LPS injection had a 380.49% rise in Nrf2 expression. HO-1 expression, which is downstream of the Nrf2 signaling pathway, was likewise 77.69% lower in the LPS group. However, compared to rats treated with LPS alone, animals treated with AVA after LPS injection showed marked increase in HO-1 expression by 279.26%.

### AVA Ameliorates LPS-induced Histopathological Alterations in the Rat Liver

**Fig. 19:**
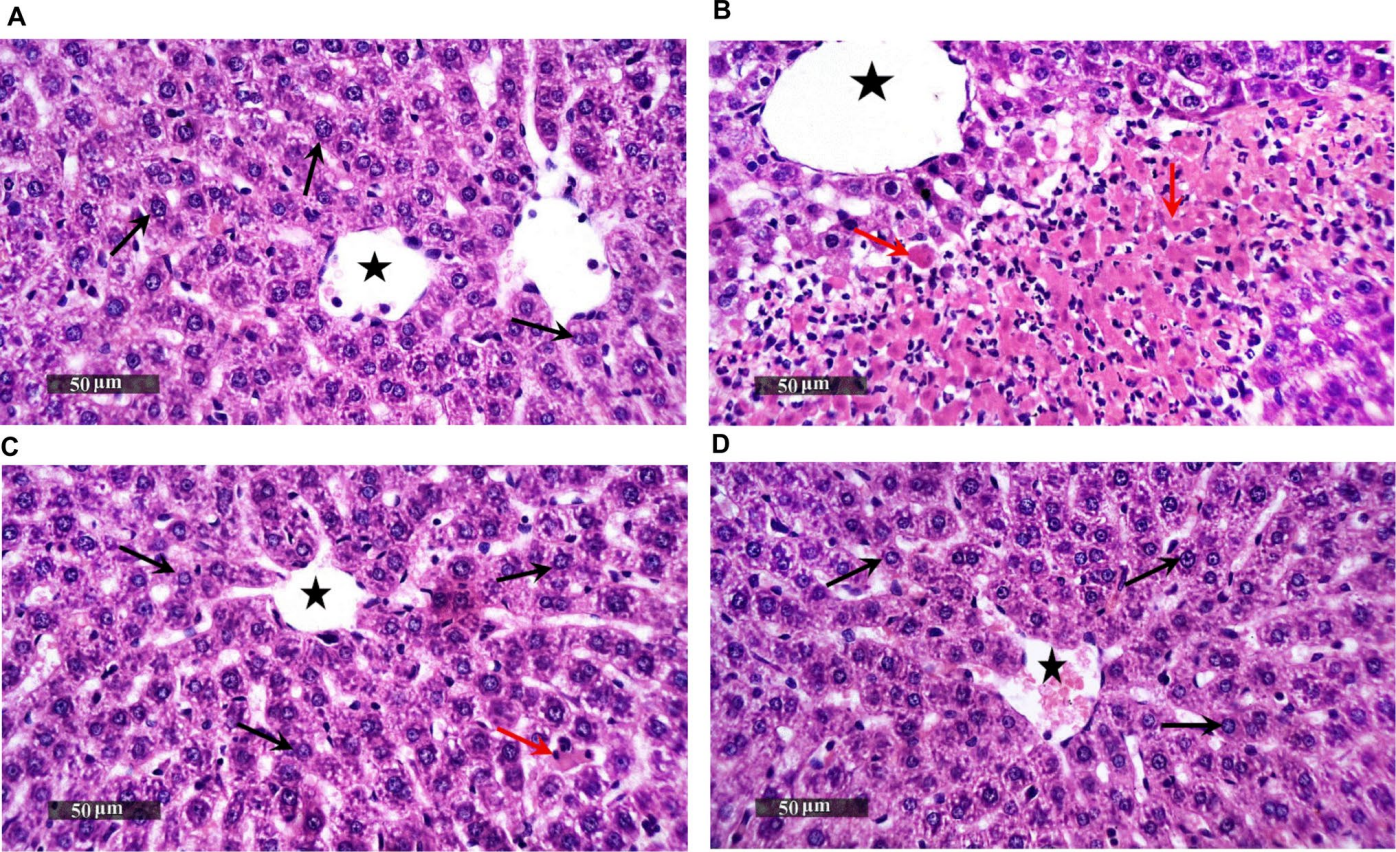
AVA improves hepatic histopathological features. Microscopic views of liver sections processed with hematoxylin and eosin staining and examined at ×200 magnification. **A** Section obtained from control group revealed a well-preserved histological organization of the hepatic parenchyma. Hepatocytes appeared abundant, intact, and orderly arranged in radiating cords, exhibiting clearly defined cell boundaries and preserved subcellular details (black arrow); minimal sporadic hepatocytes showed mild degenerative changes. Apparent intact hepatic vasculature, including sinusoids, central veins, and portal vessels, appeared patent and structurally intact (star). **B** Specimen obtained from LPS-treated group depicting reveals widespread areas of hepatocellular necrosis and cellular depress including intralobular zones invaded by mononuclear inflammatory cells infiltrates (red arrow) accompanied with moderate dilatation of hepatic vasculatures (star). **C** Section obtained from a rat treated with LPS+AVA depicting significant hepatocellular protective efficacy with remarkable figures of apparent intact hepatocytes (black arrow), minimal figures of hepatocellular necrosis, and significant amelioration of inflammatory cells infiltrates allover hepatic lobules except for few periportal areas (red arrow) and intact vasculatures (star). **D** Sections obtained from AVA group almost the same figures as normal controls. LPS, lipopolysaccharide; AVA, avanafil

As depicted in Fig. 19, liver specimens belonging to the control group revealed a well-preserved histological organization of the hepatic parenchyma. Hepatocytes appeared abundant, intact, and orderly arranged in radiating cords, exhibiting clearly defined cell boundaries and preserved subcellular details; minimal sporadic hepatocytes showed mild degenerative changes. Apparent intact hepatic vasculature, including sinusoids, central veins, and portal vessels, appeared patent and structurally intact (Fig. 19A), while those of LPS group showed multiple figures of hepatocellular necrosis and cellular depress including intralobular zones invaded by mononuclear inflammatory cells infiltrates accompanied with moderate dilatation of hepatic vasculatures (Fig. 19B). Liver sections of LPS+AVA group depicted significant hepatocellular protective efficacy with remarkable figures of apparent intact hepatocytes, minimal figures of hepatocellular necrosis, and significant amelioration of inflammatory cells infiltrates all over hepatic lobules except for few periportal areas and intact vasculatures (Fig. 19C). Liver sections of rats treated with AVA alone presented almost the same figures as normal controls, with no recorded instances of abnormal morphological changes (Fig. 19D).

## Discussion

The only second-generation PDE5I with FDA approval is avanafil. Compared to the first-generation PDE5Is, AVA has better PDE isoform selectivity and a better side effect profile [46, 47]. Experimental studies have demonstrated the protective effects of PDE5I against ischemia/reperfusion-induced tissue injury in various organs, including the myocardium [48], spinal cord [49], brain [50], and kidneys [51]. Additionally, AVA has been shown to attenuate LPS-induced acute lung injury by downregulating the TLR4/NF-κB pathway [35] and reducing MMP-9 expression [52]. Evidence suggests that PDEs influence neuronal survival, and their dysregulation may contribute to neurodegenerative and neuropsychiatric disorders, including Alzheimer’s disease, major depressive disorder, multiple sclerosis, Huntington’s disease, and Parkinson’s disease [32]. In a mouse model of LPS-induced neuroinflammation and cognitive impairment, AVA enhanced cognitive function, decreased Aβ accumulation, and attenuated inflammatory and oxidative markers [53, 54].

The gut-brain axis is increasingly recognized as a critical link between gastrointestinal and neurological health. Disruptions in gut permeability can promote the passage of bacterial endotoxins from the gut lumen into the bloodstream, triggering systemic inflammation. This inflammation plays a pivotal role in the development of both MDD and AIH [5, 6, 55, 56]. Here, the current study was designed to evaluate the efficacy of AVA in alleviating LPS-induced MDD and AIH.

Zonula Occludens-1 is a critical tight junction protein that preserves barrier integrity and supports tissue repair and cellular homeostasis [57]. In the current study, intestinal ZO-1 expression in LPS-treated rats was significantly reduced, indicating that LPS-induced inflammation compromised intestinal barrier integrity and facilitated the translocation of bacterial endotoxins into circulation. This finding is consistent with previous research indicating that a leaky gut contributes to systemic inflammation and exacerbates neuroimmune dysfunction [58]. Importantly, AVA treatment significantly increased ZO-1 expression, indicating its ability to restore intestinal barrier integrity, reduce endotoxin leakage, and improve histological alteration. Consistently, alcian blue staining revealed that LPS markedly decreased goblet cell diameter and mucin content, while AVA treatment substantially improved both parameters. These results suggest that AVA may attenuate LPS-induced intestinal barrier dysfunction.

The TLR4/NF-κB signaling pathway is well established as a key driver of LPS-induced neuroinflammation and is critically involved in the development of depression [59]. LPS stimulation activates TLR4, initiating intracellular signaling cascades that result in NF-κB activation and the subsequent release of pro-inflammatory cytokines such as TNF-α, IL-1β, and IL-6 [60]. The present findings showed that LPS exposure significantly increased TLR4 and NF-κB expression, and the inflammatory mediators TNF-α, IL-1β, and IL-6 in colon. However, AVA treatment markedly downregulated these inflammatory mediators, consistent with prior evidence showing that AVA suppressed the TLR4/NF-κB pathway and reduced inflammatory responses [30, 35].

The kynurenine pathway is a key contributor to inflammation-associated depression through the conversion of tryptophan into neurotoxic metabolites, including quinolinic acid [14, 61]. The upregulation of IDO expression in the hippocampus is directly driven by inflammatory mediators released following LPS interaction with TLR4. Moreover, activation of the TLR4/NF-κB pathway subsequently induces hippocampal IDO expression, resulting in enhanced kynurenine pathway activity and decreased serotonin availability under sustained inflammatory conditions [9, 55]. Additionally, LPS-induced inflammation compromises intestinal barrier integrity by reducing ZO-1 expression, facilitating the translocation of bacterial endotoxins into the bloodstream. This process exacerbates systemic inflammation and further promotes IDO expression. As demonstrated in our findings, LPS exposure elevated IDO expression and quinolinic acid levels. This result in agreement with [62]. However, AVA treatment was associated with reduced IDO expression and quinolinic acid levels, together with higher serotonin levels, suggesting a modulatory effect on kynurenine pathway–related alterations. Furthermore, previous studies have shown that increased MMP-9 expression is linked to BBB disruption, demyelination, inflammation, and neurotoxicity in various central nervous system (CNS) disorders. Multiple reports also indicate that MMP-9 expressions in the brain can be induced by several stimuli, including LPS [3, 63]. As shown in the current study, these changes were significantly improved following AVA treatment. Moreover, histopathological analysis confirmed the biochemical findings, showing that LPS caused severe hippocampal neuronal damage, while AVA treatment preserved neuronal structure and viability, demonstrating a strong neuroprotective effect.

Our findings also revealed that LPS administration significantly increased immobility time in the forced swimming test and reduced novel object exploration, indicating cognitive impairment and depressive-like behaviors. These behavioral changes align with previous studies, which suggest that LPS-induced systemic inflammation triggers sickness behavior and depressive symptoms [34, 64]. Notably, AVA treatment significantly reduced immobility time and improved cognitive performance, further highlighting its potential as an antidepressant through the regulation of neuroinflammatory pathway.

Although the gut-liver axis is well established in various liver diseases, its role in AIH remains less understood. Evidence indicates that AIH patients exhibit increased intestinal permeability and microbiome dysbiosis, suggesting that impaired gut barrier function and altered microbial composition may participate to the initiation and progression of the disease [7]. In the current study, administration of LPS led to marked hepatic damage, demonstrated by significantly elevated serum ALT, AST, and bilirubin levels, along with decreased albumin concentrations, increased ANA level, and notable histopathological changes. These outcomes align with earlier studies, showing that LPS-induced systemic inflammation impairs liver function and contributes to hepatic injury [20, 65–68]. Notably, AVA treatment improved hepatic function markers and reduced histopathological damage, suggesting its hepatoprotective effects. This result is in agreement with Ekor et al. [69], as sildenafil has demonstrated hepatoprotective effects by reducing serum liver enzymes and attenuating hepatic degeneration and centrilobular necrosis in paracetamol-induced hepatotoxicity model.

Moreover, the ability of AVA to downregulate expression of TLR4 in liver contributes to its protective role against LPS-induced hepatic injury. Similar hepatoprotective effects were observed in a recent study where low-dose naloxone attenuated Con A-induced AIH by modulating TLR4/NF-κB pathway [12]. This suggests that targeting these pathways may be a promising therapeutic strategy for inflammation-driven hepatic conditions.

Additionally, because increased ROS cause cellular damage and worsen neuroinflammation, oxidative stress plays a major role in the development of both AIH and MDD [70, 71]. In the current study, LPS administration significantly reduced Nrf2 and HO-1 expression in the hippocampus and liver, consistent with previous findings that oxidative stress impairs these critical antioxidant defense mechanisms [12]. Additionally, this study shows that LPS administration triggers hepatic oxidative stress, reflected by a significant rise in hepatic MDA and a clear decline in SOD and CAT activities in LPS-injected rats. The present findings indicate that AVA therapy exerts a protective effect against LPS-induced oxidative stress, as evidenced by a significant decrease in hepatic MDA and the recovery of the liver antioxidants SOD and CAT. Additionally, AVA therapy increased the levels of Nrf2 and HO-1 in the liver and hippocampus, indicating that its antioxidant qualities shield these organs from oxidative damage and injury brought on by inflammation.

Lastly, the present results show that AVA administration significantly attenuated LPS-induced behavioral, hepatic, and neuroinflammatory alterations. AVA improved intestinal barrier integrity, preserved hippocampal neuronal architecture, and restored liver function markers, indicating both neuroprotective and hepatoprotective potential. These effects appear to be mediated, at least in part, through modulation of the TLR4/NF-κB inflammatory cascade and enhancement of Nrf2/HO-1 antioxidant signaling. Collectively, these results suggest that AVA may represent a promising therapeutic candidate for managing LPS-induced MDD and AIH by targeting gut-brain-liver axis dysfunction.

## Conclusion

In conclusion, these findings highlight the multi-targeted protective effects of AVA against LPS-induced pathology by restoring gut barrier integrity, suppressing TLR4/NF-κB/IDO-mediated inflammation, and modulating the kynurenine pathway. It not only improved behavioral outcomes and cognitive performance but also attenuated hepatic injury and oxidative stress. Importantly, AVA enhanced Nrf2/HO-1 signaling in both the liver and hippocampus, reinforcing its antioxidant and cytoprotective roles. These combined effects suggest that AVA mitigates the interconnected gut-brain-liver dysfunction underlying MDD and AIH. Thus, targeting these pathways may represent a promising therapeutic strategy for inflammation-driven hepatic and neuropsychiatric disorders.

A limitation of the present study is that neurobiological analyses were confined to the hippocampus, although the prefrontal cortex is also involved in LPS-induced depressive pathology. Future studies should examine whether avanafil produces similar protective effects in the prefrontal cortex.

## Supplementary Information

Below is the link to the electronic supplementary material.

ESM 1 (JPEG 826 KB)

ESM 2 (JPEG 0.98 MB)

ESM 3 (JPEG 670 KB)

ESM 4 (JPEG 937 KB)

ESM 5 (JPEG 707 KB)

ESM 6 (JPEG 722 KB)

ESM 7 (JPEG 708 KB)

ESM 8 (JPEG 680 KB)

ESM 9 (JPEG 0.98 MB)

ESM 10 (JPEG 751 KB)

ESM 11 (JPEG 6.77 KB)

ESM 12 (JPEG 771 KB)

ESM 13 (JPEG 946 KB)

ESM 14 (JPEG 685 KB)

ESM 15 (JPEG 0.99 MB)

## Data Availability

Data will be available from the corresponding author upon reasonable request.
