# Supplementary figures and images for: Therapeutic Repurposing of Avanafil Against Lipopolysaccharide-induced Depression and Autoimmune Hepatitis: Gut-brain-liver Axis Orchestration Via Regulation of TLR4/NF-κB/IDO and Nrf2/HO-1 Pathways
Source: Mol Neurobiol. 2026 Apr 25;63(1):587. doi: 10.1007/s12035-026-05854-4 (PMC13110223; doi:10.1007/s12035-026-05854-4)

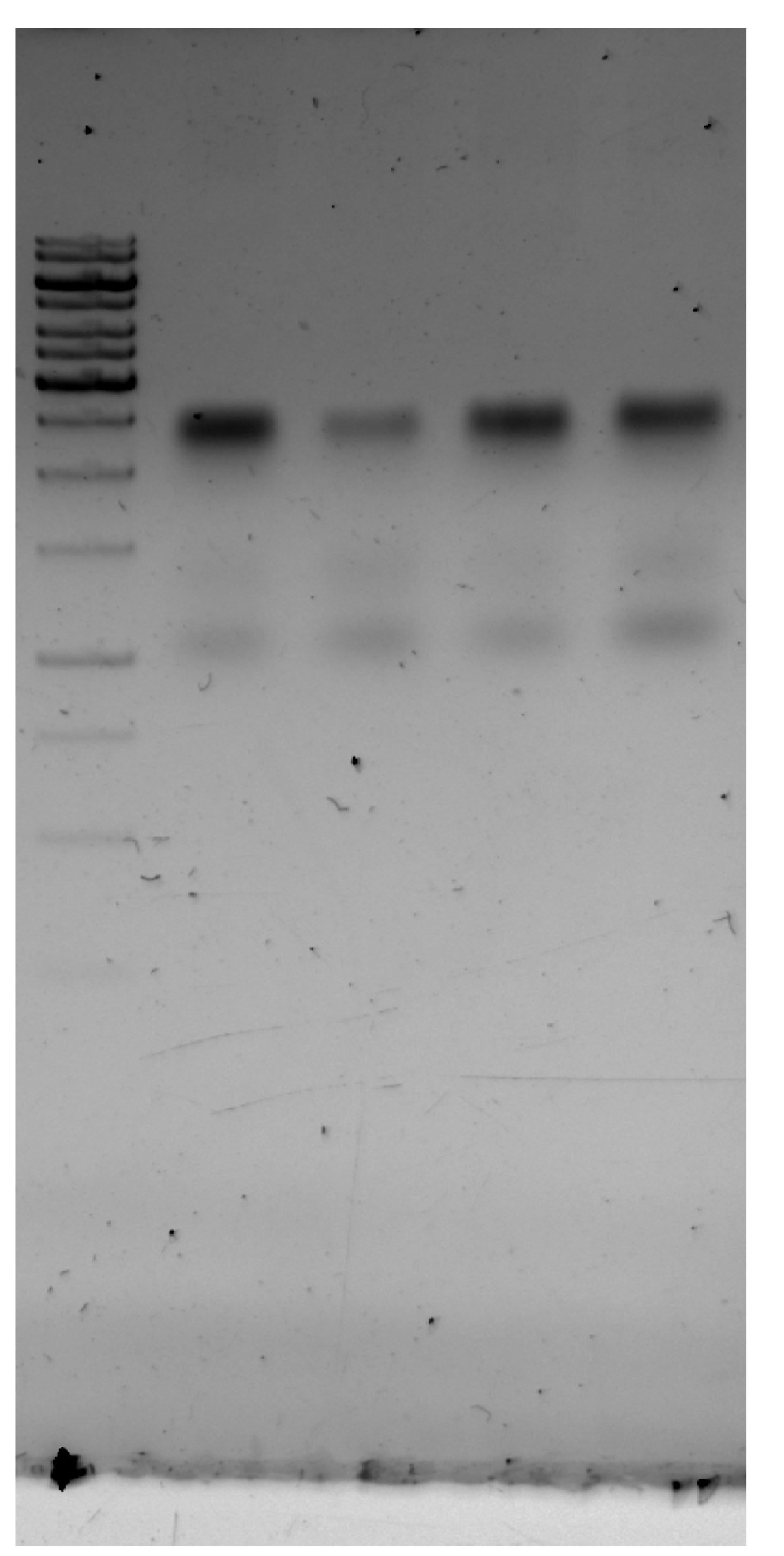

Supplement: Supplementary file 1 — (JPEG 826 KB) [file 12035_2026_5854_MOESM1_ESM.jpg]

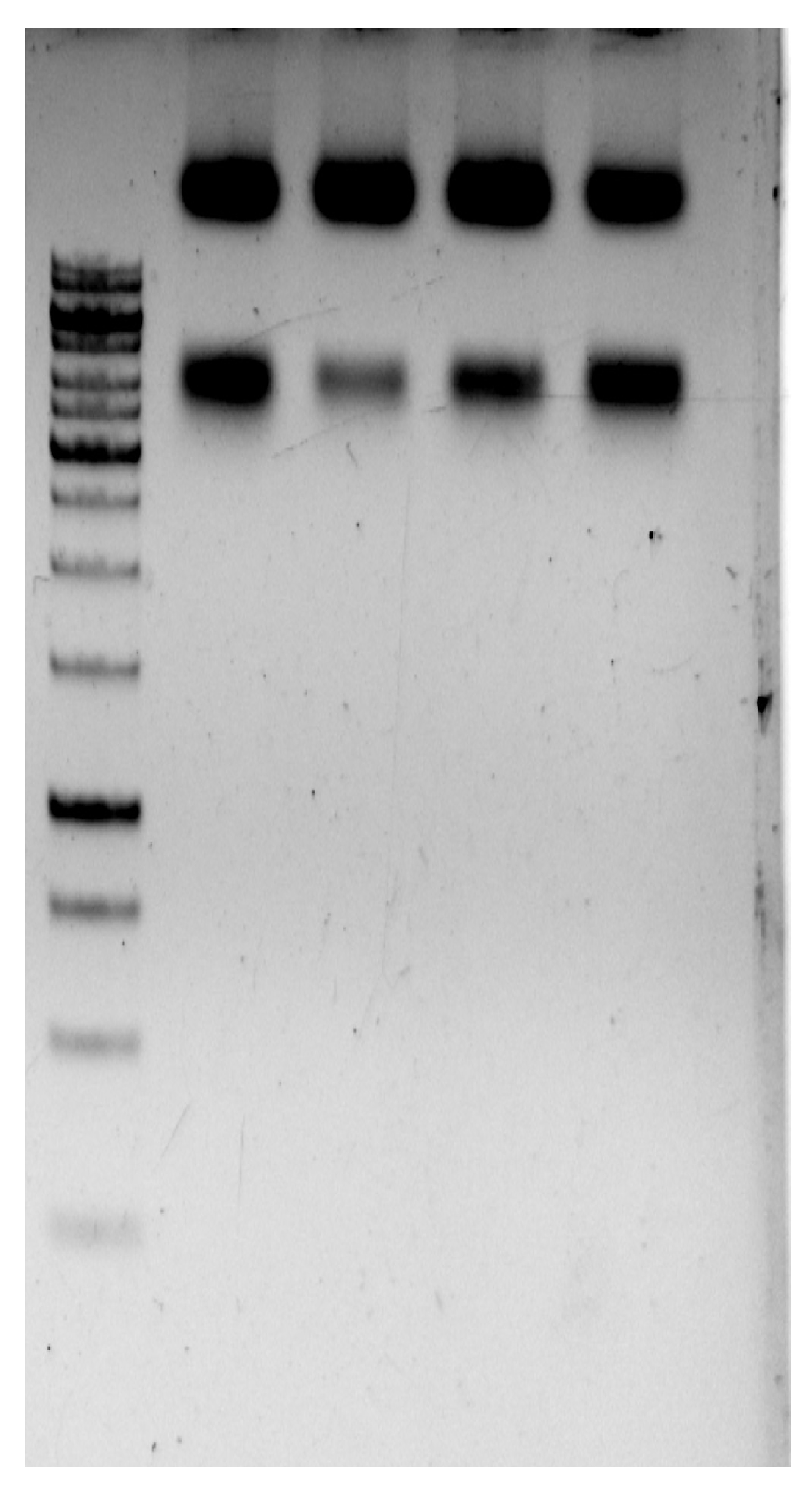

Supplement: Supplementary file 2 — (JPEG 0.98 MB) [file 12035_2026_5854_MOESM2_ESM.jpg]

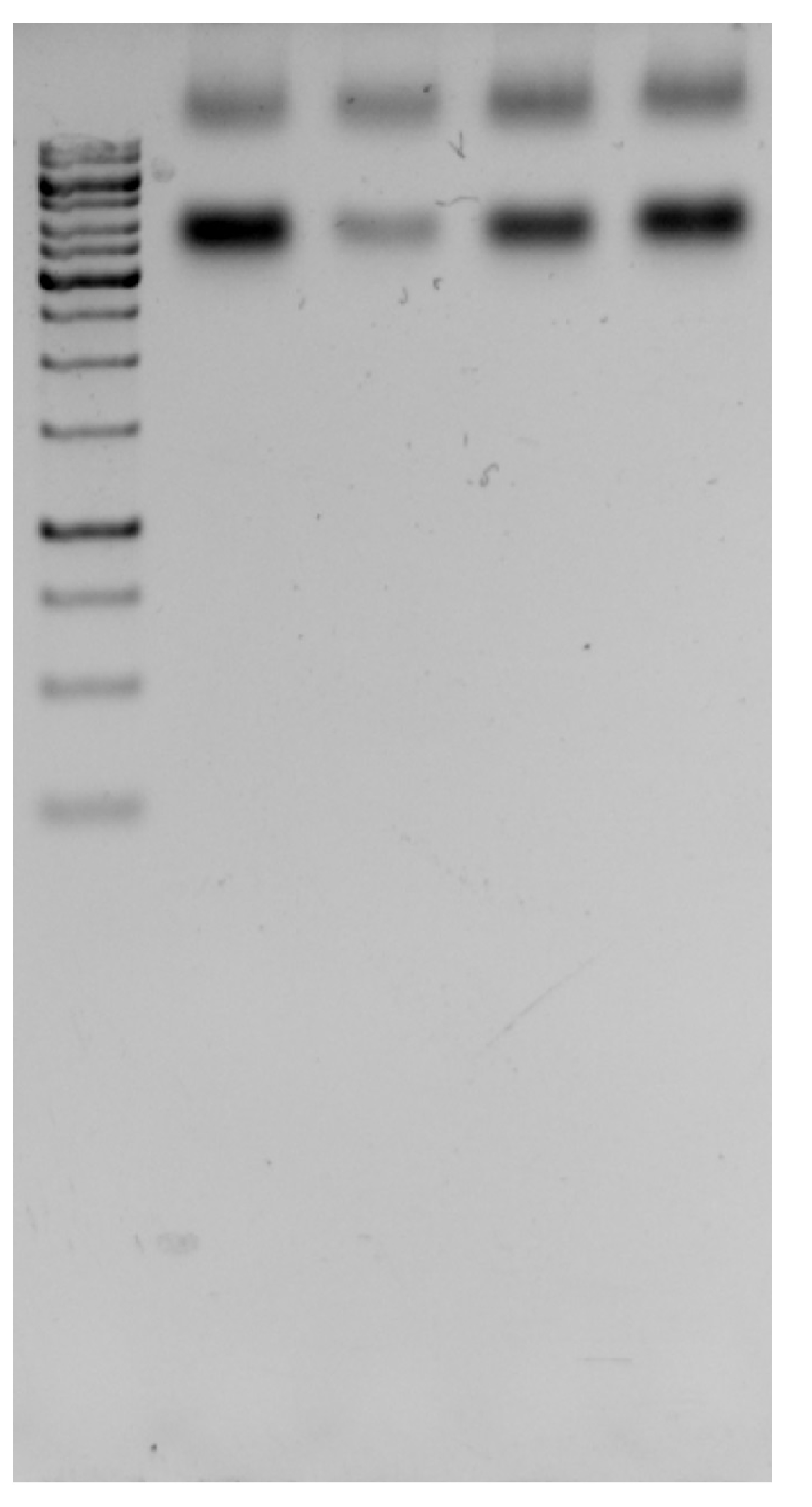

Supplement: Supplementary file 3 — (JPEG 670 KB) [file 12035_2026_5854_MOESM3_ESM.jpg]

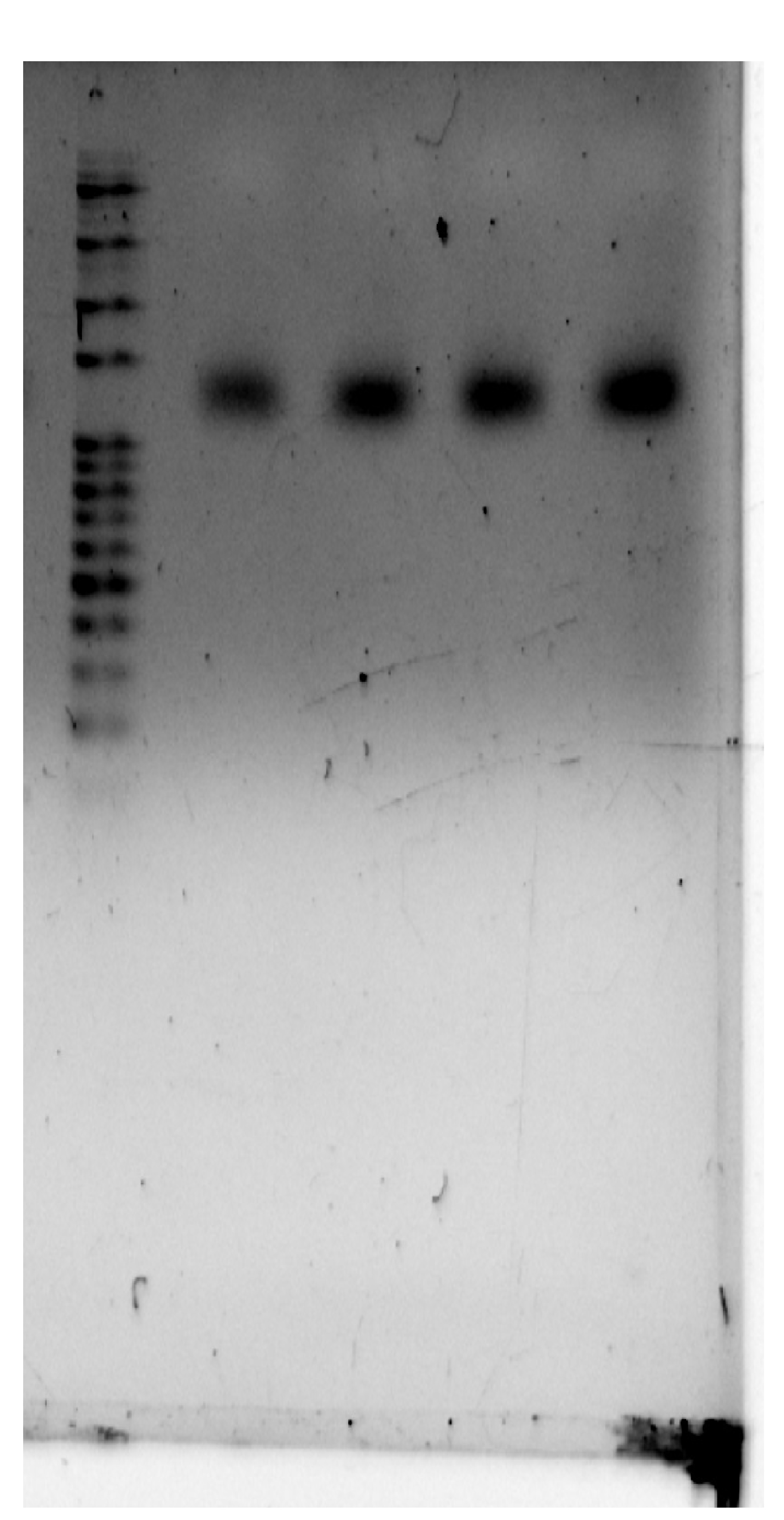

Supplement: Supplementary file 4 — (JPEG 937 KB) [file 12035_2026_5854_MOESM4_ESM.jpg]

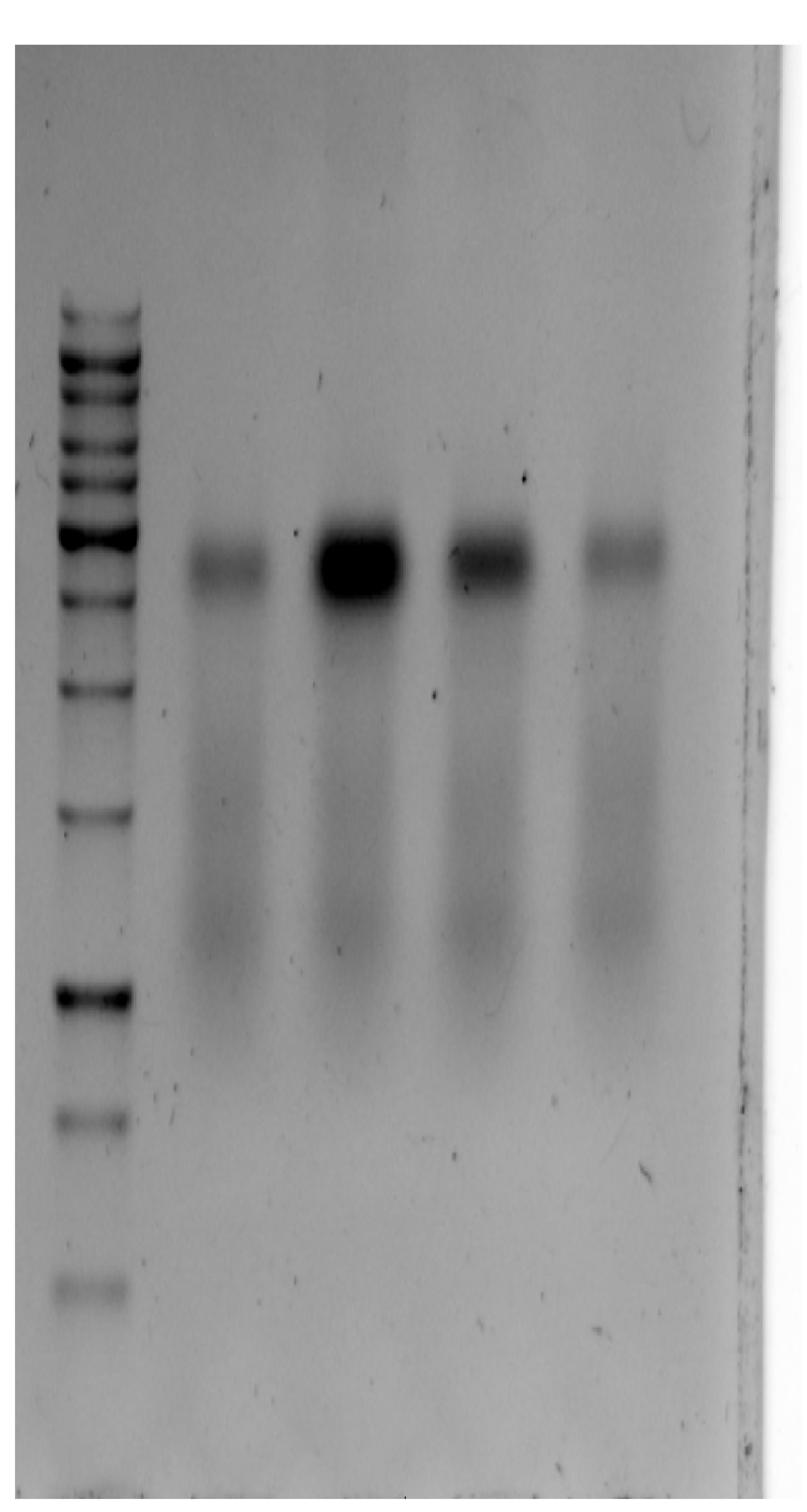

Supplement: Supplementary file 5 — (JPEG 707 KB) [file 12035_2026_5854_MOESM5_ESM.jpg]

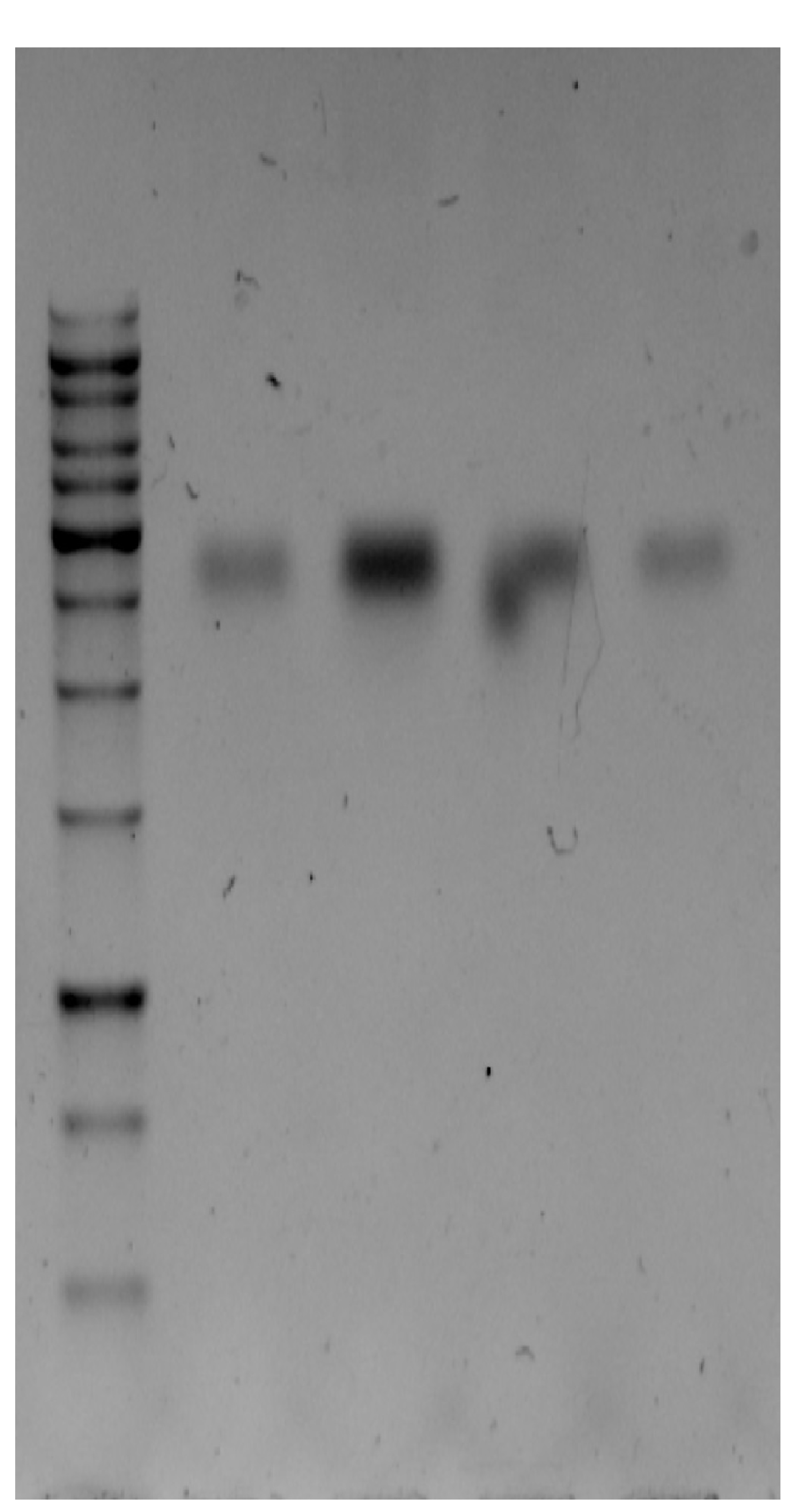

Supplement: Supplementary file 6 — (JPEG 722 KB) [file 12035_2026_5854_MOESM6_ESM.jpg]

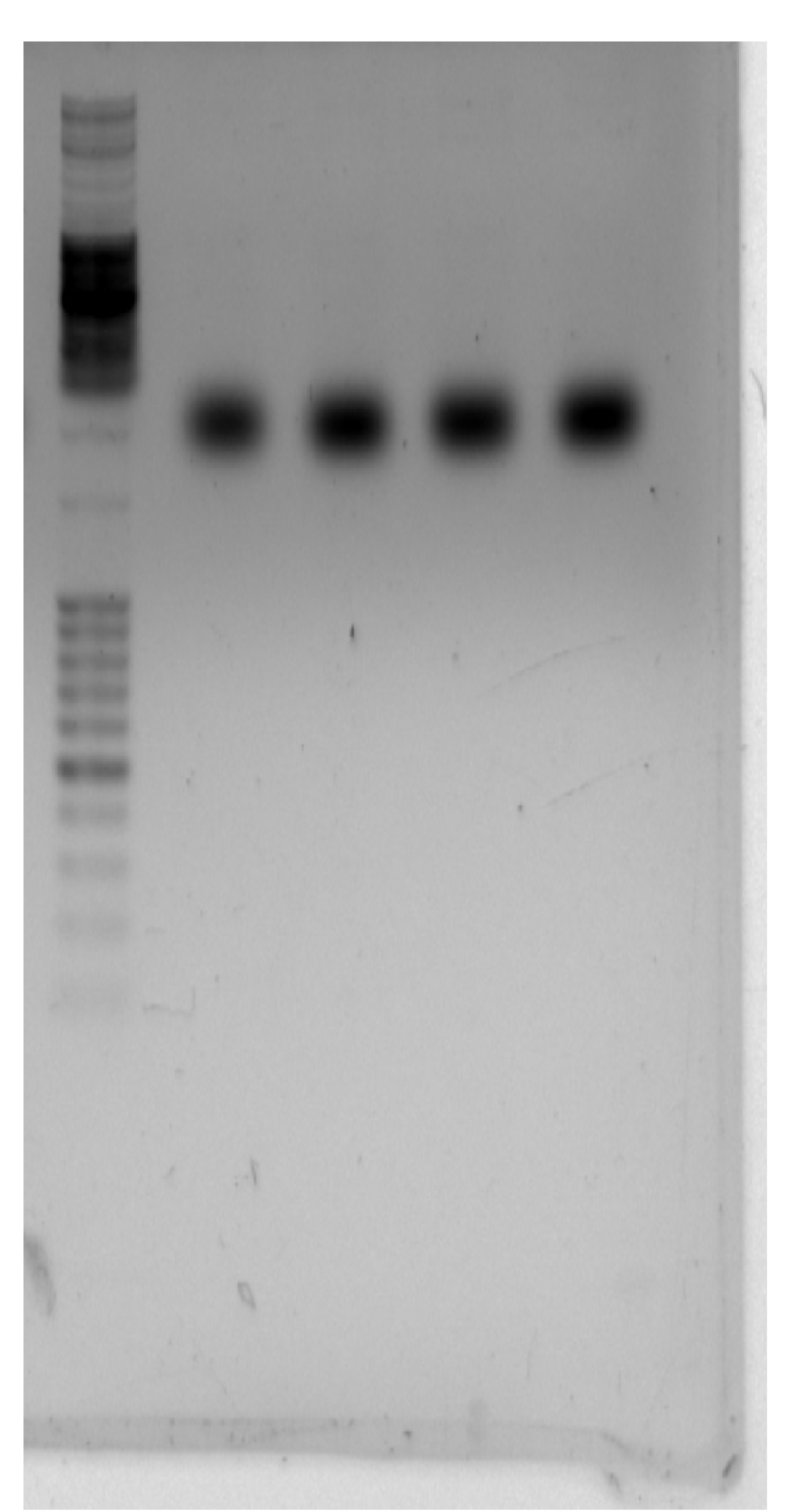

Supplement: Supplementary file 7 — (JPEG 708 KB) [file 12035_2026_5854_MOESM7_ESM.jpg]

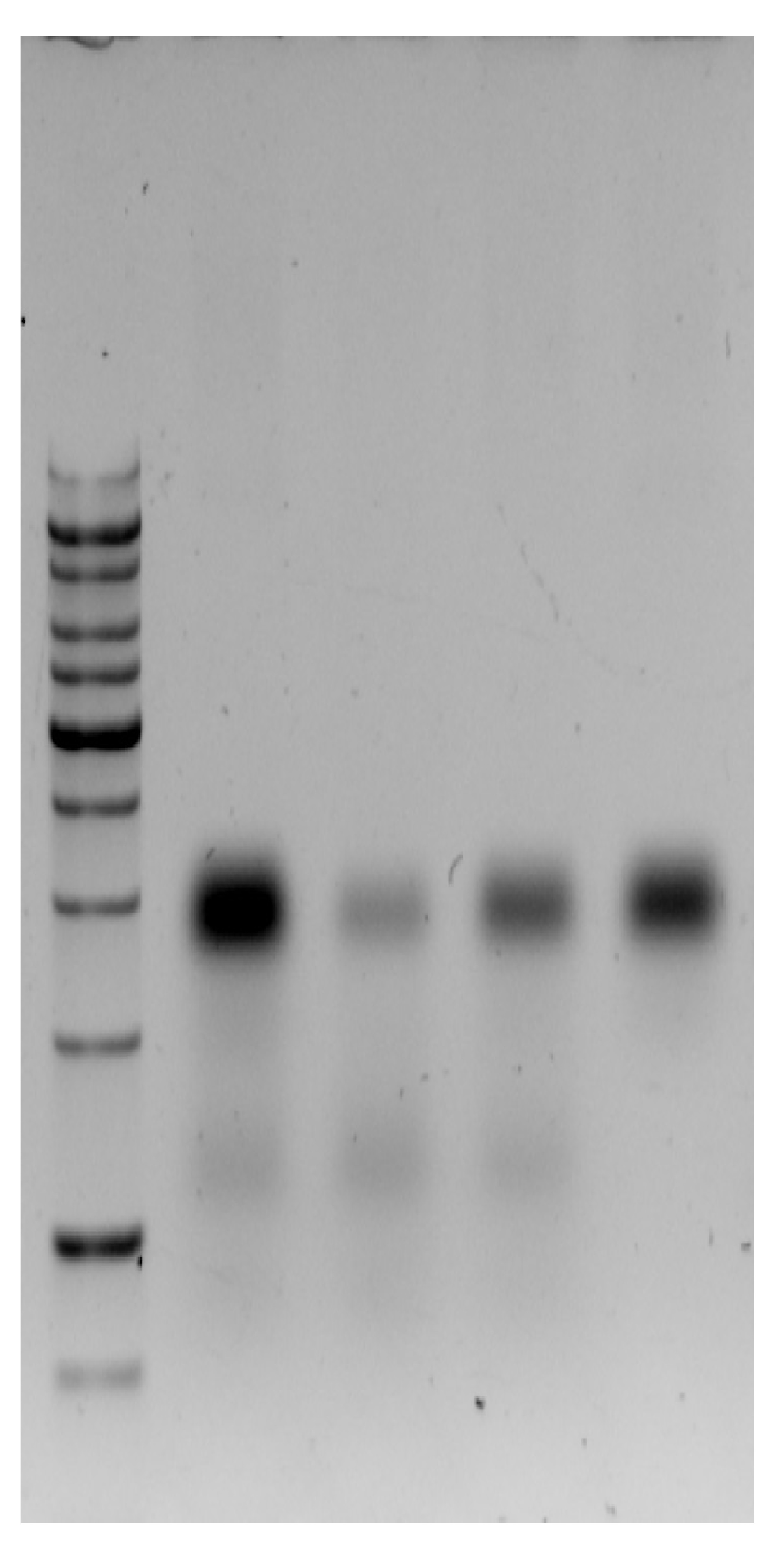

Supplement: Supplementary file 8 — (JPEG 680 KB) [file 12035_2026_5854_MOESM8_ESM.jpg]

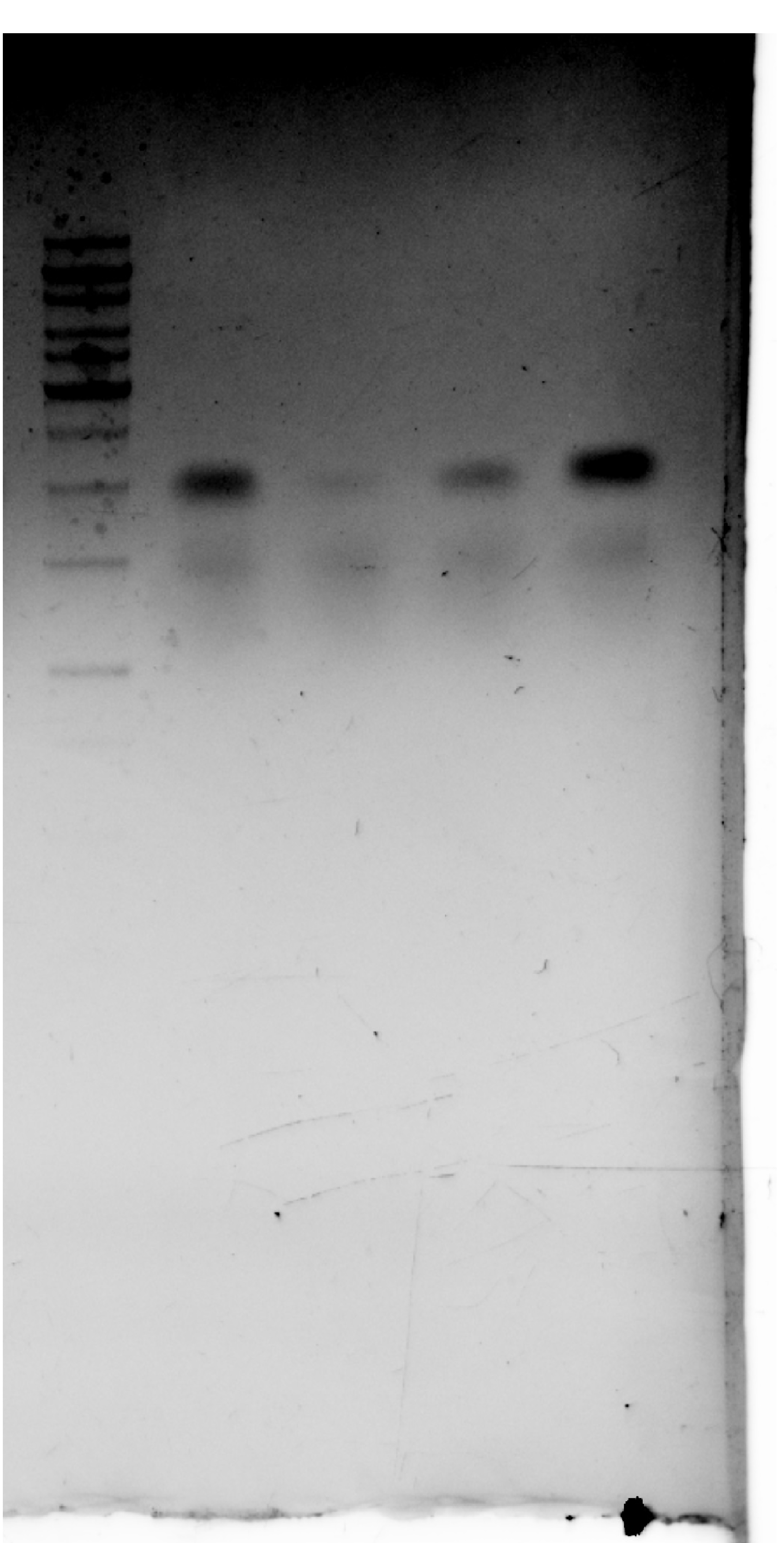

Supplement: Supplementary file 9 — (JPEG 0.98 MB) [file 12035_2026_5854_MOESM9_ESM.jpg]

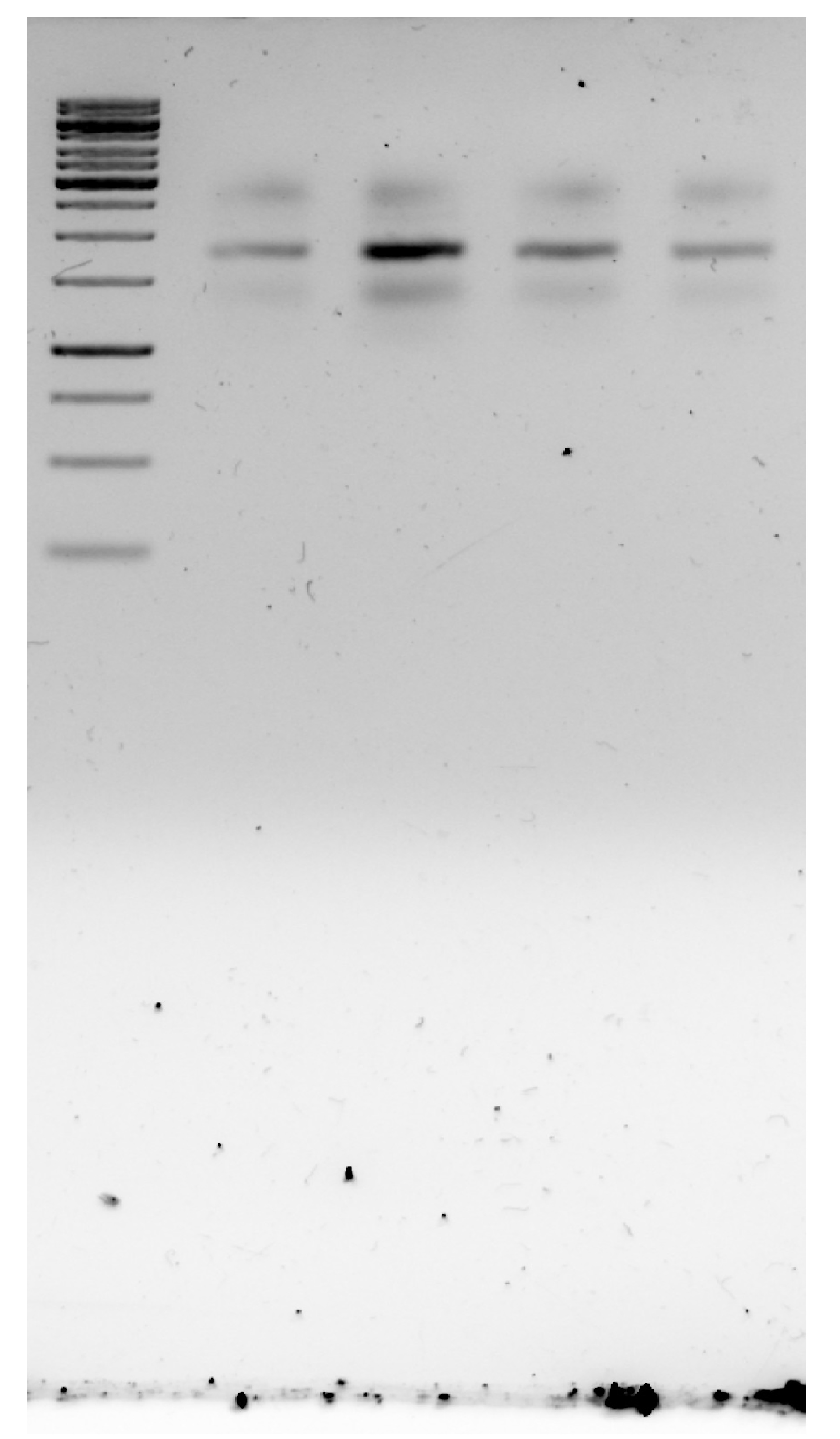

Supplement: Supplementary file 10 — (JPEG 751 KB) [file 12035_2026_5854_MOESM10_ESM.jpg]

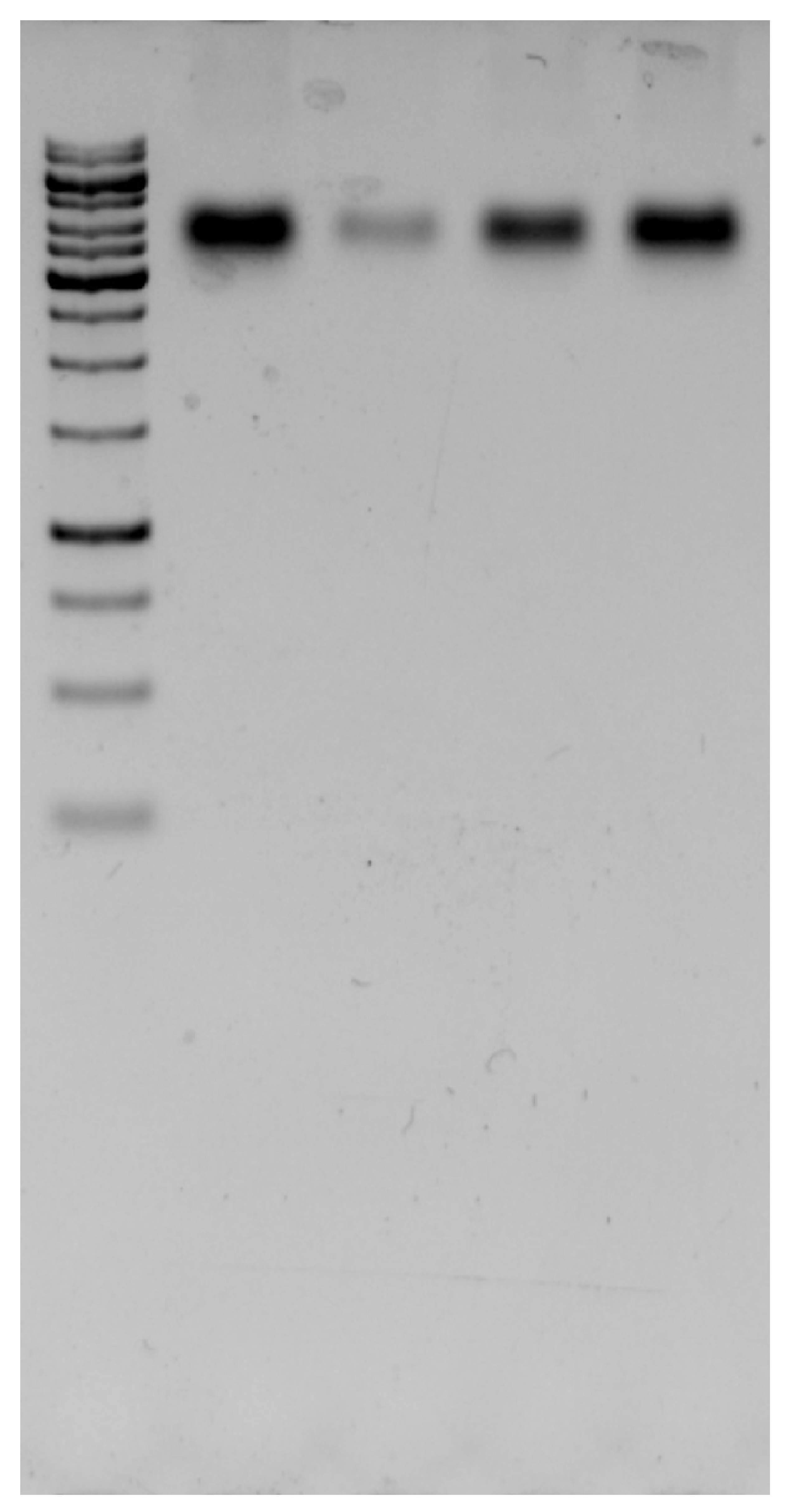

Supplement: Supplementary file 11 — (JPEG 6.77 KB) [file 12035_2026_5854_MOESM11_ESM.jpg]

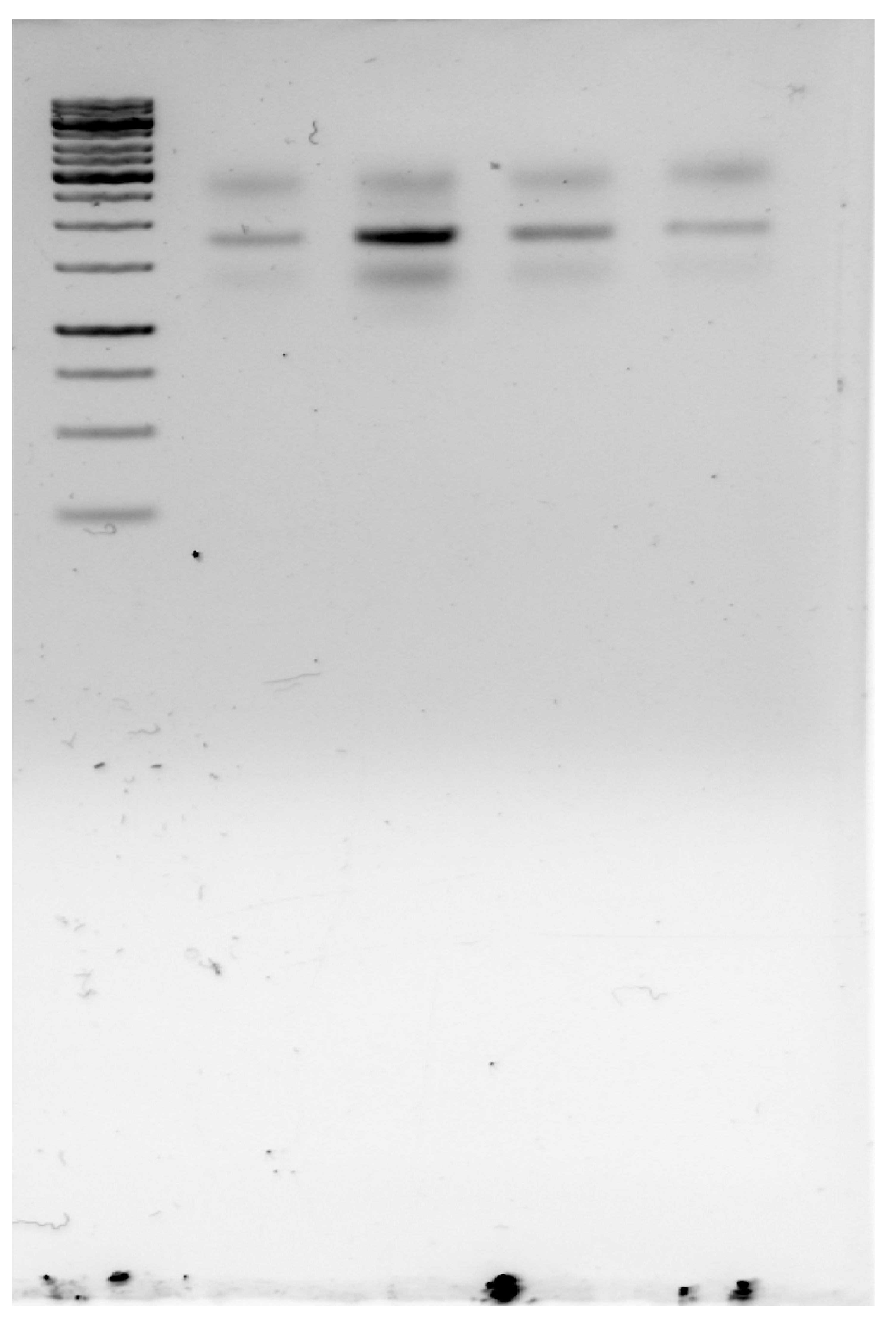

Supplement: Supplementary file 12 — (JPEG 771 KB) [file 12035_2026_5854_MOESM12_ESM.jpg]

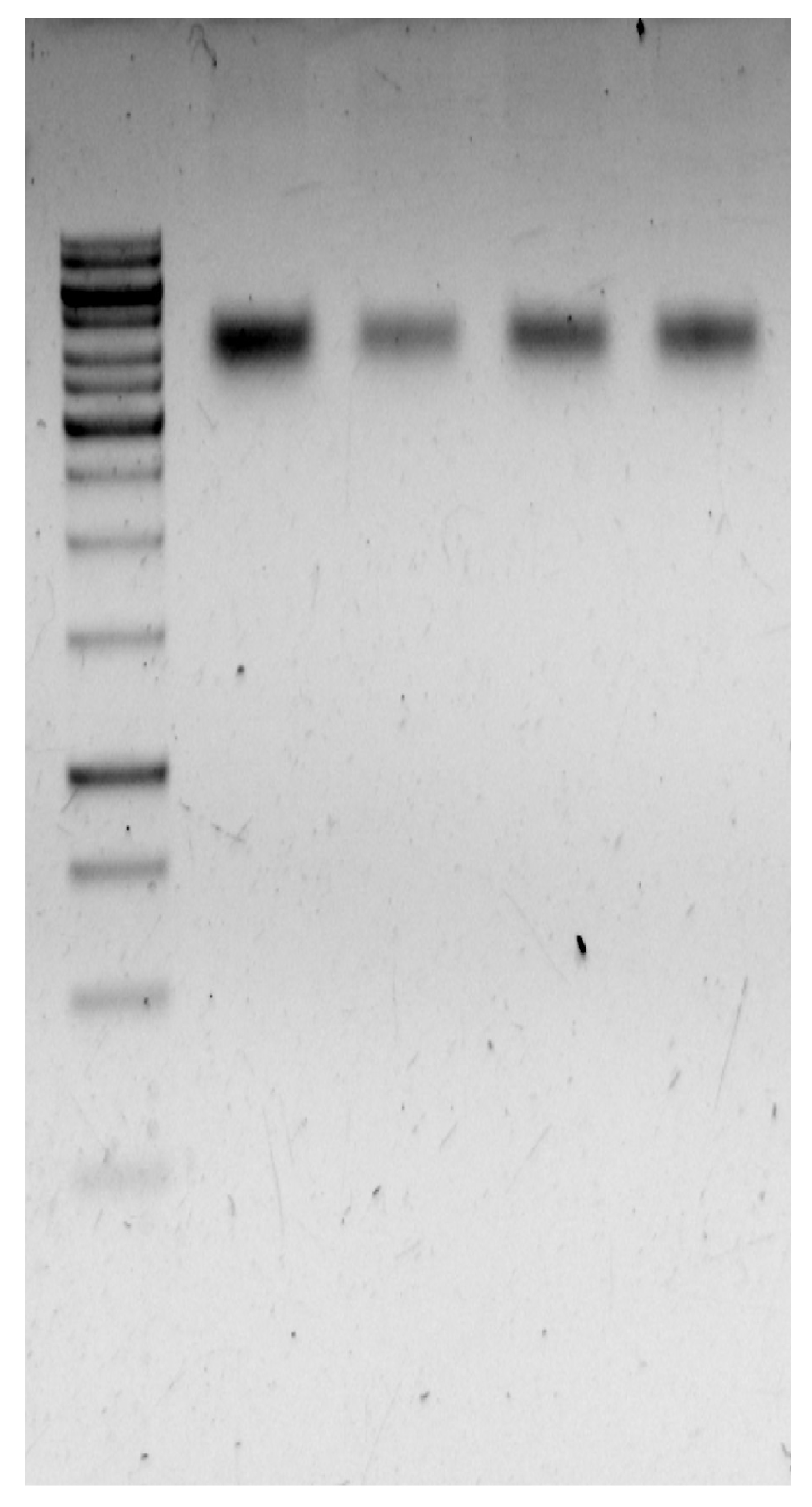

Supplement: Supplementary file 13 — (JPEG 946 KB) [file 12035_2026_5854_MOESM13_ESM.jpg]

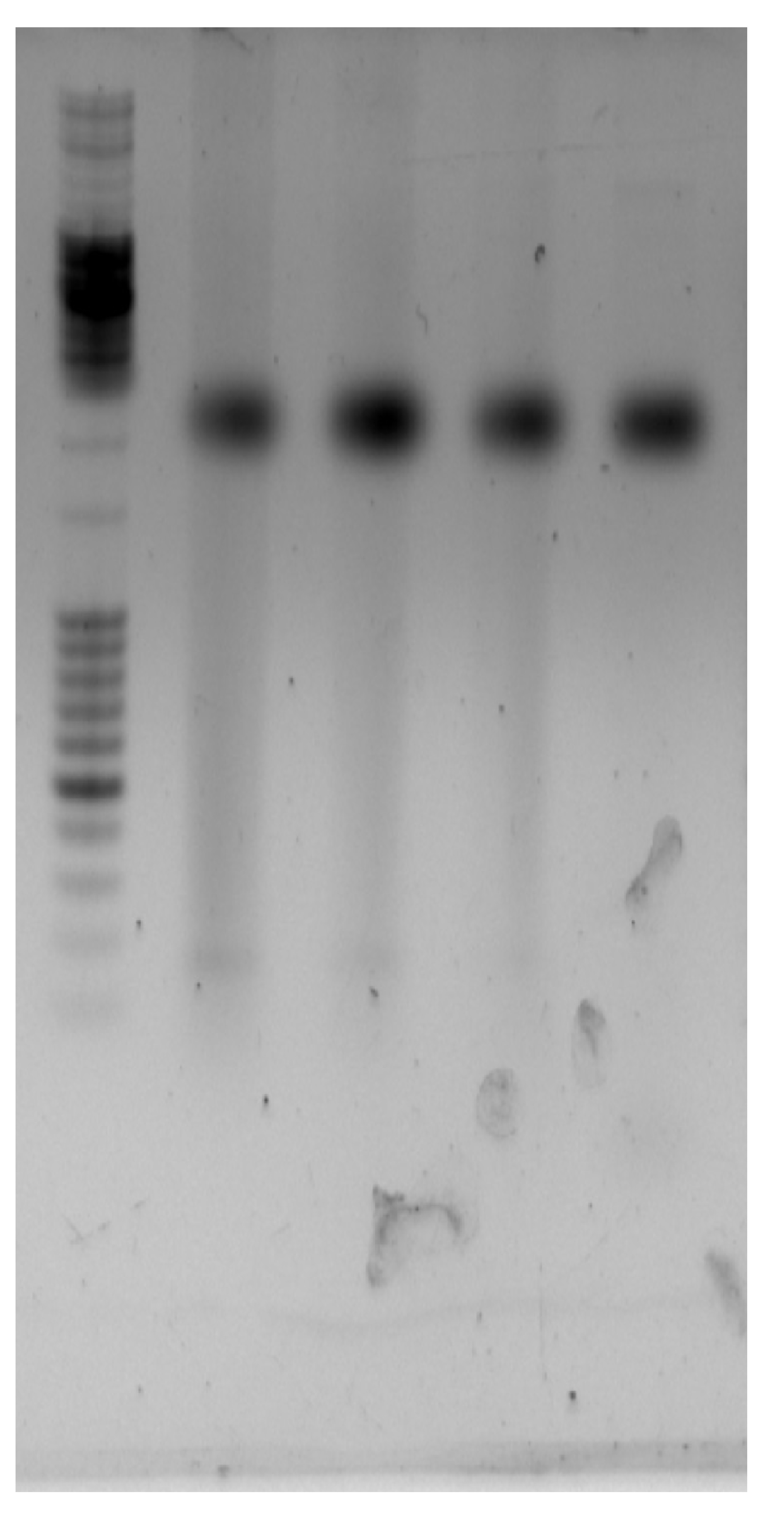

Supplement: Supplementary file 14 — (JPEG 685 KB) [file 12035_2026_5854_MOESM14_ESM.jpg]

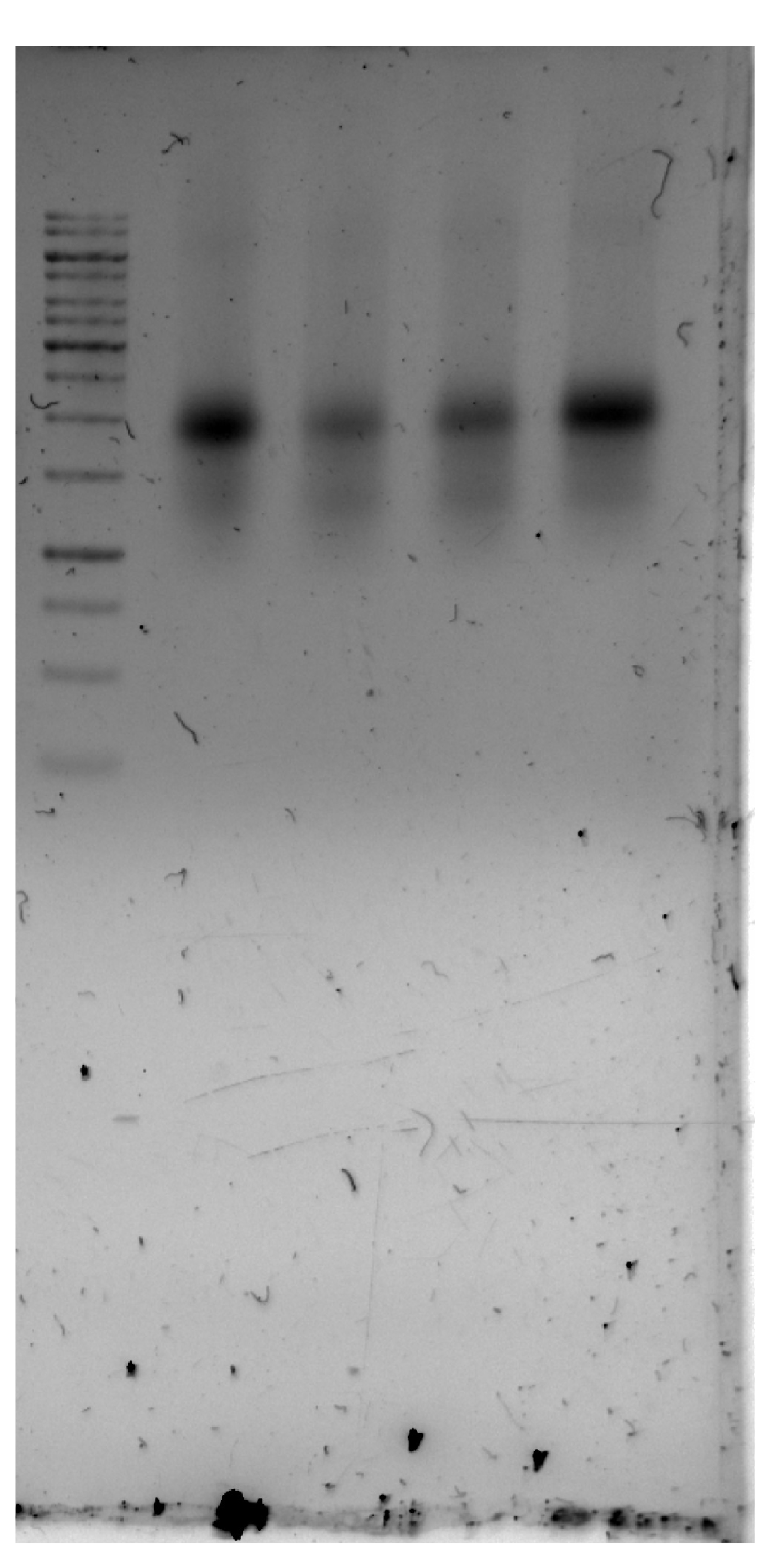

Supplement: Supplementary file 15 — (JPEG 0.99 MB) [file 12035_2026_5854_MOESM15_ESM.jpg]
